# “I Think This News Is Accurate”: Endorsing Accuracy Decreases the Sharing of Fake News and Increases the Sharing of Real News

**DOI:** 10.1177/01461672221117691

**Published:** 2022-08-21

**Authors:** Valerio Capraro, Tatiana Celadin

**Affiliations:** 1 Middlesex University London, UK; 2 University of Bologna, Italy

**Keywords:** fake news, misinformation, accuracy salience, policy making

## Abstract

Accuracy prompts, nudges that make accuracy salient, typically decrease the sharing of fake news, while having little effect on real news. Here, we introduce a new accuracy prompt that is more effective than previous prompts, because it does not only reduce fake news sharing, but it also increases real news sharing. We report four preregistered studies showing that an “endorsing accuracy” prompt (“I think this news is accurate”), placed into the sharing button, decreases fake news sharing, increases real news sharing, and keeps overall engagement constant. We also explore the mechanism through which the intervention works. The key results are specific to endorsing accuracy, rather than accuracy salience, and endorsing accuracy does not simply make participants apply a “source heuristic.” Finally, we use Pennycook et al.’s limited-attention model to argue that endorsing accuracy may work by making people more carefully consider their sharing decisions.

## Introduction

Fake news—fabricated news stories presented as if they were real (Lazer et al., 2018)—can have negative consequences for individuals and societies. Believing something that is not true might lead people to make decisions that are not in their best interest, or that are even damaging to themselves or to others (Kuklinski et al., 2000; Lazer et al., 2018; Lewandowsky et al., 2012; Pennycook & Rand, 2021).

Although fake news is not a modern phenomenon, it is becoming especially concerning in recent years because of the emergence of social media as a source of news content. As of 2020, 57% of millennials in the United States report using social media for news on a daily basis; similar percentages can be found in the United Kingdom, France, and the Netherlands, whereas they are even higher in Kenya, South Africa, and Bulgaria, where 70% of the adult population get news content from social media (Watson, 2021). Social media differ from standard media outlets because they are far less controlled and each user can potentially become a source of news content, by sharing or posting content that can potentially reach millions of other users. This lack of control makes social media especially suitable for the spread of fake news. For this reason, although fake news represents a minor proportion of the total news consumed by users (Allen et al., 2020), it is a shared opinion that finding interventions to fight fake news on social media is a problem of major importance (Kuklinski et al., 2000; Lewandowsky et al., 2012; Pennycook & Rand, 2021).

### Accuracy Salience

A set of interventions that is receiving considerable attention is rooted in the observation that some users do not share false content because they are confused or lack the knowledge or the competence to discern false headlines from true ones, but because they do not even think about whether a given news headline might be fake (Pennycook et al., 2021; Pennycook & Rand, 2021). Indeed, several works have shown that beliefs in false claims can be reduced if people are informed upfront that claims can be inaccurate (Chambers & Zaragoza, 2001; Clayton et al., 2020; Jou & Foreman, 2007; Lewandowsky et al., 2012; Schul, 1993). This suggests that making accuracy salient might make people less likely to fall for fake news. Along these lines, it has been found that having participants rate the accuracy of one unrelated headline at the outset of the study improves the extent to which participants share real news relative to fake news, both for political news (Pennycook et al., 2021) and Covid-19-related news (Epstein, Berinsky, et al., 2021; Pennycook et al., 2020; Roozenbeek et al., 2021).

In the last few years, there have been several studies testing the effect of accuracy prompts. Most prompts decrease intentions to share fake news, while keeping intentions to share real news unaffected (Epstein, Berinsky, et al., 2021; Fazio et al., 2019; Pennycook et al., 2021; Pennycook & Rand, 2021; Roozenbeek et al., 2021). See Pennycook and Rand (2022a) for a meta-analysis. There is one accuracy prompt that decreases both fake and real news sharing intentions (Jahanbakhsh et al., 2021), and one that increases real news sharing intentions, but keeps intentions to share fake news unaffected (Pennycook et al., 2020); moreover, this latter finding has not been replicated by Roozenbeek et al. (2021), who found that the same accuracy prompt decreases intentions to share fake news, while having no effect on real news. There is no known accuracy prompt that decreases fake news sharing and increases real news sharing.

### Our Contribution

We introduce a new form of accuracy prompt that is more effective than previous prompts because it both decreases intentions to share fake news and increases intentions to share real news.

We begin with a study testing two potential interventions, one that alerts participants that the headline they are reading can be fake (“Remember that it could be fake news”), and one that makes participants endorse the accuracy of the news headline (“I think this news is accurate”). On one hand, based on previous work, we expect the “fake alert” condition to decrease intentions to share fake news, while keeping intentions to share real news unaffected. On the other hand, we reasoned that endorsing accuracy might not only decrease intentions to share fake news, but also increase sharing intentions of real news, because endorsing accuracy may make people more carefully consider their sharing decisions: if you believe that a headline is accurate, then you may consider sharing it. In line with this idea, Study 1 finds that endorsing accuracy decreases intentions to share fake news and increases intentions to share real news. Moreover, it also keeps overall sharing constant. The subsequent studies are then devoted to increasing the ecological validity of Study 1 and to exploring the mechanisms through which the intervention works. Specifically, Study 2 replicates the results of Study 1 in a context where people make their sharing decisions in an environment that more closely resembles a social media newsfeed. Study 3 compares the endorsing accuracy intervention with an accuracy salience intervention that uses a wording that is very similar to the one used in the endorsing accuracy condition (i.e., “Think if this news is accurate”) and shows that the key findings are, again, specific to endorsing accuracy. Study 4 replicates the main results in a context where news sources are not displayed on the news headlines, thus suggesting that the intervention works by making people pay more attention to each news headline, rather than to the source of news more generally. Finally, we use the limited-attention model of content sharing introduced by Pennycook and colleagues (2021) to explain the results within a theoretical framework and provide more formal support for the interpretation that endorsing accuracy may work by making people more carefully consider their sharing decisions. Specifically, we approximate the function that maps the utility of sharing a piece of content to the probability of sharing it by using a logistic function, and we show that the effect of the endorsing accuracy intervention is reflected in the shape of this logistic function, which gets “closer” to the stepwise probability function, such that participants share any piece of content that gives them positive utility.

## Study 1

The aim of Study 1 is to test the effectiveness of two messages, one that alerts participants that the headline they are reading can be fake, and one that makes participants endorse the accuracy of the headline.

### Method

#### Participants

We recruited 550 U.S.-based participants on Amazon Mechanical Turk (AMT; Berinsky et al., 2012; Horton et al., 2011; Paolacci & Chandler, 2014; Paolacci et al., 2010). A sensitivity analysis showed that 550 participants are sufficient to detect an effect size of *f* = 0.129 with an α of 0.05 and power of 0.80.

#### Materials and procedure

**Figure 1.**
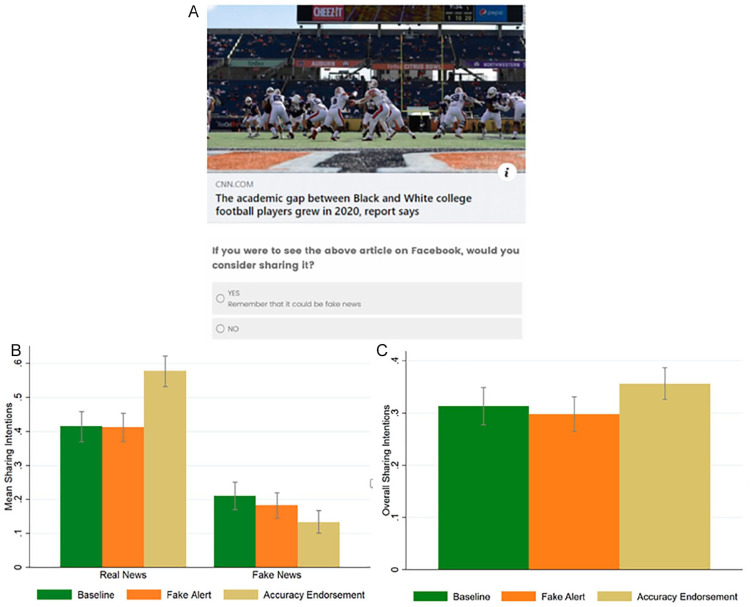
Study 1: Sample of the *fake alert* condition (A); sharing intentions split by headline veracity (*real* vs. *fake*) and condition (B); overall sharing intentions (fake and real news together) split by condition (C). *Note*. Error bars represent 95% confidence interval clustered at the participant level.

Participants were randomly divided among three conditions. In the *baseline*, participants were asked to report their intention to share each of 24 headlines (12 real, 12 fake) in Facebook format, in random order. In this and in the next studies, we used fake news collected from three news sources—dailibuzzlive.com, now8news.com, and realnewsrightnow.com—that have been classified by Melissa Zimdars (2016) as “sources that entirely fabricate information, disseminate deceptive content, or grossly distort actual news report”; and real news collected from reliable mainstream news sources (e.g., cnn.com). The headlines were collected shortly before launching the experiments. The type of news was mixed (politics, Covid-19, other). In the Supplementary Material (SM), we show that our key results are not significantly moderated by type of news. For each headline, participants were asked: “If you were to see the above article on Facebook, would you consider sharing it?” (yes/no). The *fake alert* condition differed from the baseline in that the button with the “Yes answer” to the question whether they would consider sharing the headline, contained the message: “Remember that it could be fake news” (see Figure 1A). The *accuracy endorsement* condition differed from the previous condition in that the message placed on the “Yes answer” was: “I think this news is accurate.”

#### Demographics

Before leaving the survey, participants were asked about their gender, age, and level of education.

#### Open science

The screenshots of all news headlines, as well as the data, the preregistrations, and the analysis code for this and the next studies are available online at: https://osf.io/qpw6b/?view_only=d9f0858613194fdaa2a4803029377fd8.

### Results

Figure 1B reports the average sharing intentions split by headline veracity (*real* vs. *fake*) and condition (*baseline* vs. *fake alert* vs. *accuracy endorsement*).

A linear regression with robust standard errors clustered on participants and headlines^1^ reveals that the interaction between the *real news* dummy and the *accuracy endorsement* condition dummy is significant (*b* = 0.239, *p* < .001; *t* = 6.06, 95% confidence interval [CI] [0.162, 0.317]) while the interaction between the *real news* dummy and the *fake alert* condition dummy is not significant (*b* = 0.026, *p* = .411, *t* = 0.82, 95% CI [−0.036, 0.087]). Moreover, the coefficients of the two interactions (*real news* × *fake alert* vs. *real news* × *accuracy endorsement*) are statistically different, *F*(1, 13194) = 36.76, *p* < .001. Regression tables can be found in the SM, Table S1. Post hoc analyses show that participants in the *accuracy endorsement* condition tend to share more real news compared with both the *baseline* and the *fake alert* conditions (*accuracy endorsement* vs. *baseline: b* = 0.163, *p* < .001; *accuracy endorsement* vs. *fake alert: b* = 0.165, *p* < .001), whereas there is no difference between the sharing intentions of real news in the *baseline* vs. the *fake alert* condition, *b* = −0.002, *p* = .937. Participants in the *accuracy endorsement* condition tend to share less fake news compared with both the *baseline* and the *fake alert* conditions (*baseline* vs. *accuracy endorsement: b* = −0.077, *p* = .006; *fake alert* vs. *accuracy endorsement: b* = −0.049, *p* = .044), while there is no difference between the sharing intentions of fake news in the *baseline* vs. the *fake alert* condition, *b* = −0.028, *p* = .336. Regarding overall sharing (fake and real news together), we find that there is no difference between the sharing intentions in the *accuracy endorsement* condition and the *baseline* (*b* = 0.043, *p* = .227), and between the *fake alert* condition and the *baseline* (*b* = −0.015, *p* = .539), while there is a marginally significant difference between the *accuracy endorsement* condition and the *fake alert* condition (*b* = 0.058, *p* = .068) (see Figure 1C). In the SM, Table S1, we also show that these results are not significantly moderated by type of news (political, COVID-19, other).

**Table 1.** Estimated Logistic Parameters in Studies 1 to 3.

	Fake alert/accuracy salience		Accuracy endorsement	
Study	θ	μ	θ	μ
Study 1	1.14 (±0.07)	1.30 (±0.13)	2.17 (±0.08)	0.85 (±0.15)
Study 2	0.55 (±0.07)	2.90 (±0.13)	1.18 (±0.08)	1.61 (±0.15)
Study 3	0.92 (±0.08)	1.87 (±0.13)	1.50 (±0.08)	1.35 (±0.15)

*Note.* θ is equal to the coefficient of the real news dummy in the logistic regression; therefore, the error in brackets is simply the standard error returned by the regression. μ is computed by dividing the coefficient of the constant by the coefficient of the real news dummy, with a minus in front; the error in the bracket is defined as the sum of the standard error of the coefficient and the constant.

In sum, Study 1 reveals that endorsing accuracy decreases intentions to share fake news, increases intentions to share real news, and keeps overall sharing constant. For these reasons, it seems to be a far more promising intervention than the fake alert message.

## Study 2

Study 2 aims at increasing the ecological validity of the previous study.

### Methods

#### Participants

*N* = 558 participants were recruited on AMT. A sensitivity analysis showed that 558 participants are sufficient to detect an effect size of *f* = 0.125 with an α of 0.05 and power of 0.80.

#### Materials and procedure

**Figure 2.**
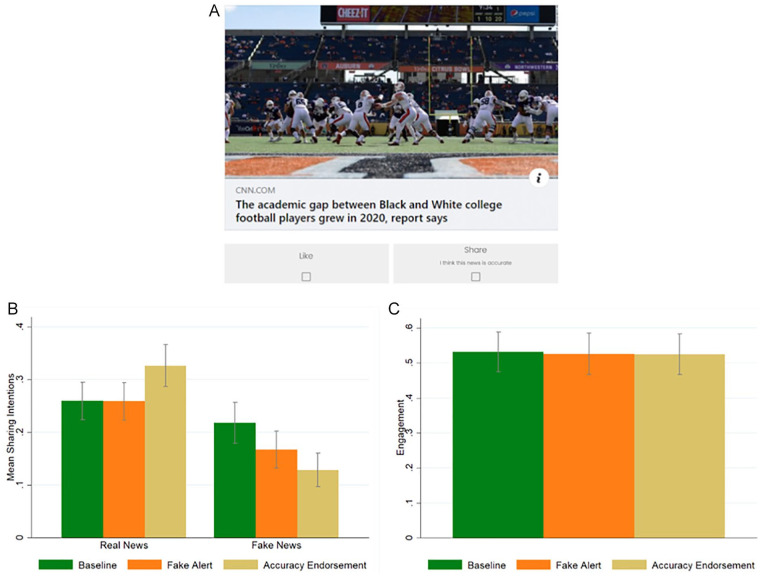
Study 2: Sample of the *accuracy endorsement* condition (A); sharing intentions split by headline veracity (real vs. fake) and condition (B); overall engagement, defined as (likes + shares)/number of news headlines, split by condition (C). *Note*. Error bars represent 95% CI clustered at the participant level.

The design was similar to Study 1, but participants, instead of being asked to report the intention to share one news headline at a time, one for each screen, could scroll through the news headlines in a similar fashion as they do in social media such as Facebook or Twitter. Moreover, instead of being asked, for each news headline, whether they would consider sharing it, below each news headline, we included two buttons: a “like button” and a “share button.” Responses were not forced, and multiple answers were allowed. In other words, participants were free to scroll through the newsfeed and, for each headline, they could like it, share it, do both, or neither (see Figure 2A). As in Study 1, participants were randomly divided among three conditions: *baseline*, *fake alert*, and *accuracy endorsement*.

#### Demographics

Before leaving the survey, participants were asked about their gender, age, and level of education.

### Results

Figure 2B reports the average sharing intentions split by headline veracity (*real* vs. *fake*) and condition (*baseline* vs. *fake alert* vs. *accuracy endorsement*).

A linear regression with robust standard errors clustered on participants and headlines reveals that the interactions between the *real news* dummy and the two condition dummies are both significant (*real news* × *fake alert: b* = 0.050, *p* = .019, *t* = 2.35, 95% CI [0.008, 0.092]; *real news* × *accuracy endorsement: b* = 0.156, *p* < .001, *t* = 6.19, 95% CI [0.107, 0.206]). Regression tables can be found in the SM, Table S2. The coefficients of the two interactions (*real news* × *fake alert* vs. *real news* × *accuracy endorsement*) are statistically different, *F*(1, 13386) = 18.48, *p* < .001. Post hoc analyses find that participants in the *accuracy endorsement* condition tend to share more real news compared with both the *baseline* and the *fake alert* conditions (*accuracy endorsement* vs. *baseline: b* = 0.067, *p* = .013; *accuracy endorsement* vs. *fake alert: b* = 0.068, *p* = .013), whereas there is no difference between the sharing intentions of real news in the *baseline* vs. the *fake alert* condition, *b* = −0.001, *p* = .975. Participants tend to share less fake news, compared with the *baseline*, both in the *fake alert* and in the *accuracy endorsement* conditions (*baseline* vs. *fake alert: b* = −0.051, *p* = .043; *baseline* vs. *accuracy endorsement: b* = −0.089, *p* < .001); there is a marginally significant difference between the sharing of fake news in the *accuracy endorsement* condition with respect to the *fake alert* condition, *b* = −0.039, *p* = .089. Regarding overall (real and fake news) sharing, we find no difference across treatments (*baseline* vs. *accuracy endorsement: b* = −0.011, *p* = .682; *fake alert* vs. *accuracy endorsement: b* = 0.015, *p* = .549; *fake alert* vs. *baseline*, *b* = −0.026, *p* = .270). Finally, we compare the overall engagement, that we define as the total number of reactions (number of likes plus number of shares) divided by the number of news headlines, across conditions. We find that there is no difference in the overall engagement across treatments (*baseline* vs. *fake alert: b* = −0.006, *p* = .892; *baseline* vs. *accuracy endorsement: b* = −0.007, *p* = .876; *fake alert* vs. *accuracy endorsement: b* = −0.001, *p* = .977) (see Figure 2C). In the SM, Table S2, we also show that these results are not significantly moderated by type of news (political, COVID-19 related, other) and headline display order (see Table S11). In the SM, Table S3, we also report the analysis of the liking intentions. We found no significant differences across conditions.

In sum, Study 2 confirms the broad finding of Study 1 that endorsing accuracy is a promising intervention, as it decreases intentions to share fake news, increases intentions to share real news, while keeping overall sharing and overall engagement (likes + shares) constant.

## Study 3

One limitation of the first two studies is that the endorsing accuracy condition differs from the fake alert condition in dimensions other than the act of endorsing. Specifically, the endorsing accuracy condition explicitly uses the term “accuracy,” while the fake alert condition uses the term “fake news.” Therefore, it is possible that the effects of endorsing accuracy are not due to the act of *endorsing* accuracy, but to the way in which the concept of accuracy is made salient. To address this point, in Study 3 we compare the endorsing accuracy condition with an accuracy salience condition that uses a message that is very similar to the one used in the endorsing accuracy condition.

### Method

#### Participants

*N* = 550 participants were recruited on AMT. A sensitivity analysis showed that 550 participants are sufficient to detect an effect size of *f* = 0.129 with an α of 0.05 and power of 0.80.

#### Materials and procedure

**Figure 3.**
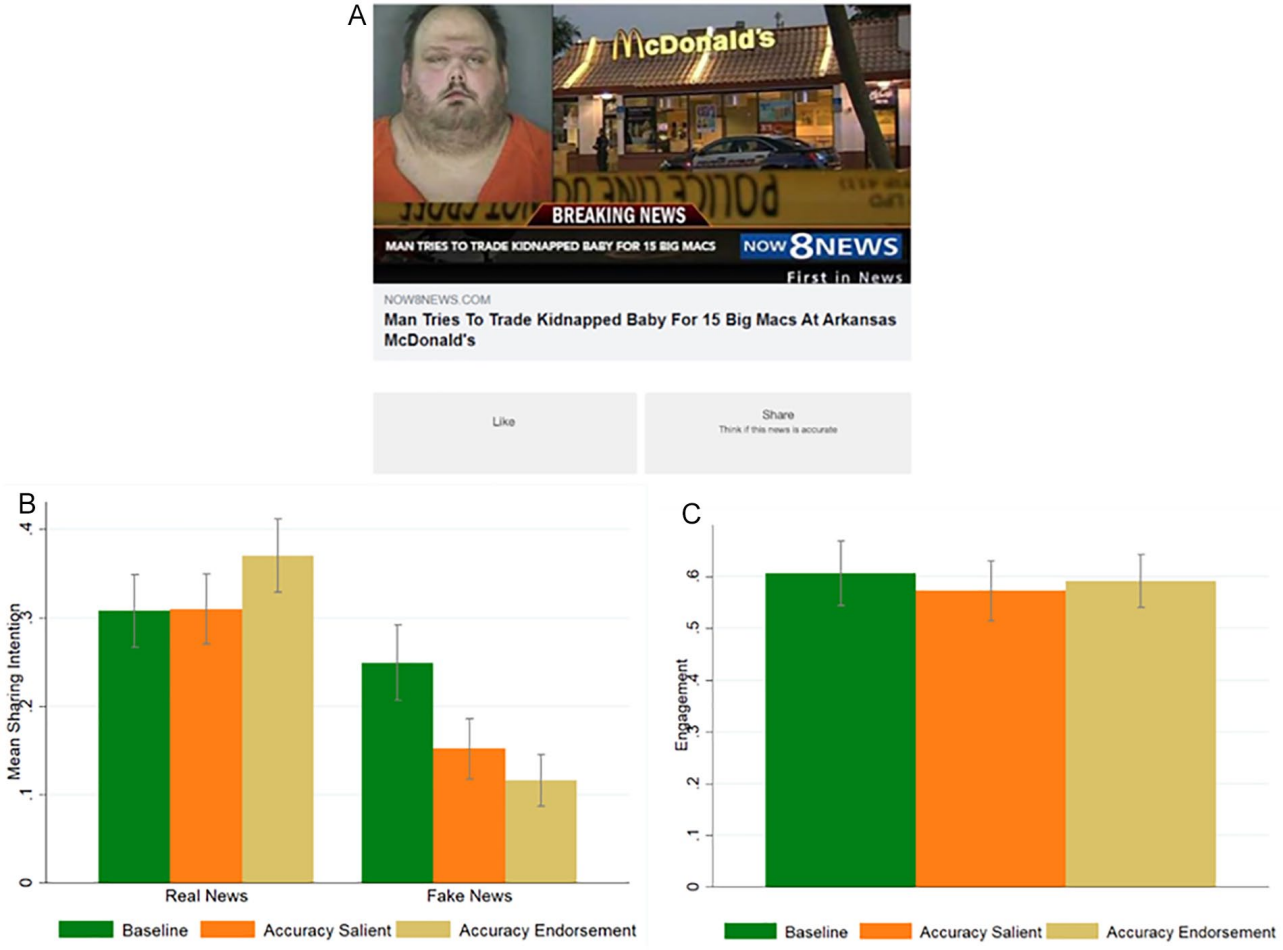
Study 3: Sample of the *accuracy salient* condition (A); sharing intentions split by headline veracity (*real* vs. *fake*) and condition (B); overall engagement, defined as (likes + shares)/number of news headlines, split by condition (C). *Note*. Error bars represent 95% CI clustered at the participant level.

Participants were randomly divided among three conditions: *baseline*, *accuracy salience*, and *accuracy endorsement*. The *baseline* and the *accuracy endorsement* conditions were identical to those of Study 2; the *accuracy salience* condition was similar to the *accuracy endorsement* condition, but the message displayed into the sharing button was: “Think if this news is accurate.” As in Study 2, participants could scroll through the news headlines and, for each of them, they could like it, share it, do both, or neither. We also slightly changed the visualization of the available answers to eliminate the “squares” in the “like” and “share” buttons in Study 2 (see Figure 3A).

#### Demographics

Before leaving the survey, participants were asked about their gender, age, and level of education.

### Results

Figure 3B reports the average sharing intentions split by headline veracity (*real* vs. *fake*) and condition (*baseline* vs. *accuracy salience* vs. *accuracy endorsement*).

A linear regression with robust standard errors clustered on participants and headlines reveals that the interactions between the *real news* dummy and the two condition dummies are both significant (*real news* × *accuracy salience: b* = 0.099, *p* < .001, *t* = 3.57, 95% CI [0.045, 0.154]; *real news* × *accuracy endorsement: b* = 0.196, *p* < .001, *t* = 6.22, 95% CI [0.131, 0.258]). Regression tables can be found in the SM, Table S4. The coefficients of the two interactions (*real news* × *accuracy salience* vs. *real news* × *accuracy endorsement*) are statistically different, *F*(1, 13194) = 12.23, *p* < .001. Post hoc analyses reveal that participants in the *accuracy endorsement* condition tend to share more real news compared with both the *baseline* and the *accuracy salience* conditions—although the difference between the *accuracy endorsement* condition and the *baseline* was, this time, marginally significant (*accuracy endorsement* vs. *baseline: b* = 0.063, *p* = .056; *accuracy endorsement* vs. *accuracy salience: b* = 0.061, *p* = .026)—whereas there is no difference between the sharing intentions of real news in the *baseline* vs. the *accuracy salience* condition, *b* = 0.002, *p* = .948. Participants tend to share less fake news, compared with the baseline, both in the *accuracy salience* and in the *accuracy endorsement* conditions (*baseline* vs. *accuracy salience: b* = −0.097, *p* < .001; *baseline* vs. *accuracy endorsement: b* = −0.133, *p* < .001), whereas there is no difference in the sharing intentions of fake news between the *accuracy endorsement* condition and the *accuracy salience* condition, *b* = −0.036, *p* = .110. Regarding overall sharing, we find that in the *accuracy endorsement* condition this was not different from the overall sharing in the *baseline* (*b* = −0.035, *p* = .269), while the overall sharing in the *accuracy salience* condition was marginally significantly smaller than in the *baseline* (*b* = −0.048, *p* = .077), although not significantly different from the *accuracy endorsement* condition, *b* = 0.012, *p* = .591. Finally, we find that there is no difference in the overall engagement across conditions (*accuracy salience* vs. *baseline: b* = −0.035, *p* = .443; *accuracy endorsement* vs. *baseline: b* = −0.015, *p* = .740; *accuracy salience* vs. *accuracy endorsement: b* = 0.019, *p* = .631) (see Figure 3C). In the SM, Table S4, we also show that these results are not significantly moderated by type of news (political, COVID-19 related, other) and headline display order (see Table S11). In the SM, Table S5, we also report the analysis of the liking intentions. We found no significant differences across conditions.

In sum, Study 3 replicates the broad finding of Studies 1 and 2 that endorsing accuracy decreases intentions to share fake news, increases intentions to share real news, while keeping overall sharing and overall engagement constant. Moreover, it shows that a message similar to the endorsing accuracy message, but which only makes accuracy salient, without endorsing it, fails to increase intentions to share real news, suggesting that it is the act of endorsing that increases intentions to share real news, and not accuracy salience per se.

## Study 4

In the previous studies, the source of news was displayed on the news headline. We made this design choice to present headlines in the same format as they are presented on social media. However, this raises a theoretical question regarding the channel through which endorsing accuracy works: does endorsing accuracy make people pay more attention to the accuracy of the headlines, or does it make them more likely to apply a more general “source heuristic” to determine which news to share (e.g., known vs. unknown news source)? In this study, we answer this question.

### Method

#### Participants

*N* = 372 participants were recruited on AMT. A sensitivity analysis showed that this sample size is sufficient to detect an effect size of *f* = 0.140 with an α of 0.05 and power of 0.80.

#### Materials and procedure

**Figure 4.**
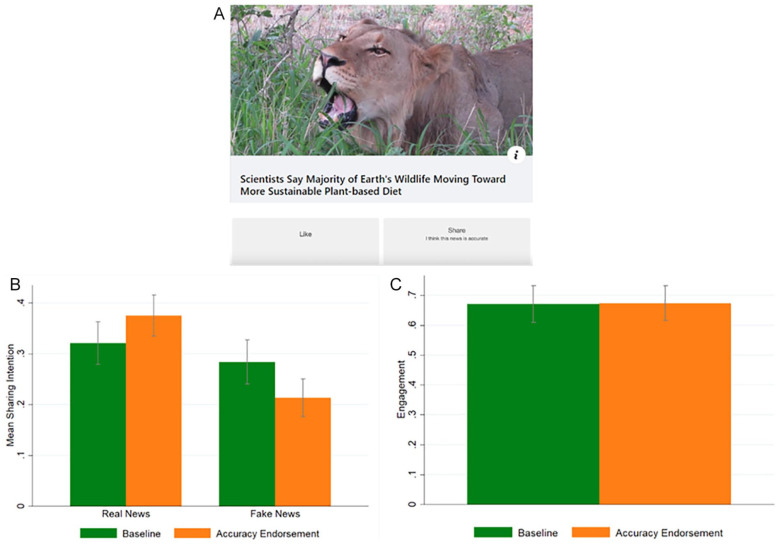
Study 4: Sample of the *accuracy endorsement* condition (A); sharing intentions split by headline veracity (*real* vs. *fake*) and condition (B); overall engagement, defined as (likes + shares)/number of news headlines, split by condition (C). *Note*. Error bars represent 95% CI clustered at the participant level.

Participants were randomly divided between two conditions: *baseline* and *accuracy endorsement*. The *baseline* and the *accuracy endorsement* conditions were identical to those of the previous studies, but the source of news was removed from the news headlines (see Figure 4A).

#### Demographics

Before leaving the survey, participants were asked about their gender, age, and level of education.

### Results

Figure 4B reports the average sharing intentions split by headline veracity (*real* vs. *fake*) and condition (*baseline* vs. *accuracy endorsement*).

A linear regression with robust standard errors clustered on participants and headlines reveals that the interaction between the *real news* dummy and the condition dummy is significant (*b* = 0.126, *p* < .001, *t* = 5.00, 95% CI [0.076, 0.175]). Regression tables can be found in the SM, Table S6. Participants in the *accuracy endorsement* condition tend to share more real news (*b* = 0.055, *p* = .049) and less fake news (*b* = −0.071, *p* = .009) compared with the baseline. Regarding overall sharing and overall engagement, we find no difference between conditions (overall sharing: *b* = −0.008, *p* = .791; overall engagement: *b* = .004, *p* = .933) (see Figure 4C). In the SM, we also show that these results are not significantly moderated by type of news (political, COVID-19 related, other; see Table S6) and headline display order (see Table S11). In the SM, Table S7, we also report the analysis of the liking intentions. We found no significant differences across conditions.

In sum, Study 4 replicates the broad finding of the previous studies—that endorsing accuracy increases real news sharing, decreases fake news sharing, while having little effect on overall sharing and overall engagement—in a context in which news sources are not displayed, thus suggesting that endorsing accuracy makes people pay attention to the accuracy of each headline, rather than making them apply a general source heuristic to decide which headline to share.

## Explaining the Effect of Endorsing Accuracy Using Pennycook et al.’s (2021) Model of Content Sharing

In this section, we show that the effect of endorsing accuracy can be explained by the “limited-attention” model of content sharing introduced by Pennycook and colleagues (2021). Specifically, we make formal our interpretation that “endorsing accuracy may work by making people more carefully consider their sharing decisions,” and we test one of its predictions using the utility function proposed by Pennycook and colleagues.

Pennycook and colleagues’ model rationalize participants’ decision to share a piece of content *x* through a utility function *U*(*x*) *=* −*a*_1_*b*_1_*F*(*x*) *+ a*_2_*b*_2_*C*_2_(*x*) *+ . . . + a_k_b_k_C_k_*, where *F* represents whether the content is fake, *C*_2_, . . ., *C_k_* are other dimensions that might be important at the moment of sharing (e.g., partisan alignment, humorousness, etc.), the *a_i_*’s are context-dependent parameters that represent whether dimension *i* is salient (*a_i_* = 1) or not (*a_i_* = 0) at the moment of sharing, and the *b_i_*’s are individual, context-independent parameters representing the weight a person assigns to dimension *i*. In standard utility theory, *a_i_* = 1, for all *i*. Pennycook and colleagues’ “limited-attention” model assumes that people have cognitive constraints that make them unable to consider all the dimensions together; that is, not all *a_i_*’s are equal to 1. However, a given dimension can be made salient using nudges. In particular, the role of accuracy salience is to turn *a*_1_ = 1. Then, participants share *x* with some probability *p*(*U*(*x*)), which is an increasing function of *U*(*x*). Ideally, *p* would be a stepwise function such that (assuming that the utility of non-sharing is zero) a participant shares any piece of content that gives them positive utility. However, in more realistic situations, *p* could be a logistic function: *p*(*share*) *=* (1 *+ e*^-θ(U(x)-µ)^)^-1^, where μ represents the value of *U*(*x*) at which the person is equally likely to share and to not share, and θ represents the steepness of the transition from sharing to not sharing (Pennycook et al., 2021). This logistic function approximates the stepwise function for μ *→* 0 and θ → ∞.

Studies 1 to 3 show that endorsing accuracy does something more than simply making accuracy salient. Since *a*_1_ is already turned to 1 by accuracy salience and since all the other parameters in Pennycook et al.’s utility function are either context-independent or accuracy-unrelated, the only possibility is that accuracy endorsement affects the probability function *p* that maps the utility of sharing to the probability of sharing. Specifically, if our interpretation that endorsing accuracy works by strengthening the link between the utility of sharing and the sharing decision is correct, then we should observe that, compared with accuracy salience, the estimated μ gets closer to 0, while the estimated θ increases; in other words, we should observe that the effect of endorsing accuracy, compared with accuracy salience, is to make the logistic function “closer” to the stepwise function. Here we test this prediction using the data collected in Studies 1 to 3.

In Studies 1 and 2, we compare the estimates in the fake alert condition with those in the endorsing accuracy condition, while in Study 3 we compare the estimates in the accuracy salience condition with those in the endorsing accuracy condition. To do so, we consider the utility function *U*(*x*) *= b*_1_(−*F*(*x*)), obtained from the Pennycook et al.’s utility function, assuming (a) *a*_1_ = 1, because the concept of accuracy is salient in the fake alert, the accuracy salience, and the endorsing accuracy conditions^2^; (b) the role played by the other dimensions *C*_2_,*. . ., C_k_* does not change across conditions, because the headlines are the same across conditions, and these dimensions are not related to accuracy. Note that −*F*(*x*) is the *real news* dummy, so, to estimate θ and μ, we need to run, for each of the relevant conditions, a logistic regression predicting sharing intentions as a function of the *real dummy*: the coefficient of the *real news* dummy will be equal to θ, while μ will be equal to minus the coefficient of the constant divided by the coefficient of the *real* dummy. Table 1 reports the estimated μ and θ for Studies 1 to 3. The errors in brackets are computed as follows: the error of θ is simply the standard error returned by the logistic regression; the error of μ is the sum of the errors of the two coefficients. It is easily seen that, in line with the prediction, in all studies, the θ in the fake alert/accuracy salience conditions are always larger than the corresponding θ in the accuracy endorsement condition. Similarly, the μ in the fake alert/accuracy salience conditions are always smaller (and closer to zero) than the corresponding μ in the accuracy endorsement condition.^3^

## Discussion

We tested three potential interventions to fight the spread of fake news on social media: (a) an alert into the sharing button, with written “Remember that it could be fake news,” decreased intentions to share fake news in Study 2, but not in Study 1, and left intentions to share real news unaffected in both studies; (b) an accuracy endorsement into the sharing button, “I think this news is accurate,” decreased intentions to share fake news, increased intentions to share real news, and kept overall sharing and engagement (likes + shares) unaffected; (c) an accuracy prompt into the sharing button, “Think if this news is accurate,” decreased intentions to share fake news, while keeping intentions to share real news unaffected.

These results suggest that adding a short message into the sharing button on social media, which makes users endorse the accuracy of the corresponding headline, can decrease fake news sharing and increase real news sharing, while having little effect on overall sharing and engagement. This intervention has several positive sides. It is as invasive as other interventions already in use on social media; indeed, Facebook and Twitter have already implemented interventions that tag each news headline regarding vaccines with a message and a link, or with pop-ups that alert users who are sharing without reading (Clark, 2021; Vincent, 2020). Furthermore, an endorsing accuracy intervention can be easily implemented on social media: when the social media algorithm recognizes a post as a news headline, it may automatically add the endorsing accuracy message into the sharing button. Finally, compared with previous interventions based on accuracy salience, which largely operate by reducing sharing of fake news (Pennycook & Rand, 2022a), endorsing accuracy also increase sharing of real news. This is crucial, because increasing exposure to accurate information is as important, if not more so, than reducing exposure to false information (Acerbi et al., 2022).

In Studies 2 to 4, we also explored whether our accuracy prompts affect liking intentions. We found no effect. To the best of our knowledge, there is only work exploring the effect of accuracy prompts on liking intentions: Epstein, Sirlin, et al. (2021) found that rating the accuracy of each headline does improve also liking discernments of those headlines. The main difference between our design and that of Epstein and colleagues is that our accuracy prompt is displayed only into the sharing button, and not into the liking button, and this might be the reason why we observed an effect only on sharing intentions.

Recently, scholars have stressed the importance of clarifying by which mechanism an intervention works (Pennycook et al., 2021; Pennycook & Rand, 2022b; Roozenbeek et al., 2021). From a theoretical perspective, we could rationalize our findings by building upon the limited-attention model of content sharing introduced by Pennycook et al. (2021). Specifically, we argued that endorsing accuracy does not simply make the concept of accuracy salient, but it also makes people more carefully consider their sharing decisions. Formally, this means that the logistic function that maps the utility of sharing a piece of content to the probability of sharing it gets “closer” to the stepwise probability function, such that a participant shares each content that gives them a positive utility.

Our results have some limitations. First, we measured only intentions to share (Study 1) or sharing decisions in an environment that simulated a social media newsfeed (Studies 2–4). Although this is an improvement with respect to previous research, which typically measured only sharing intentions, it is still limited; for example, our simulated newsfeed is not visually identical to the Facebook newsfeed; moreover, we only added the “like” button and not the other reactions. However, we believe that this is not a major limitation, as previous work found that sharing intentions collected on AMT are correlated to actual sharing decisions on Twitter (Mosleh et al., 2020) and because our Studies 2 to 4 gave results that were broadly consistent with Study 1. Second, our experiments were conducted on AMT with U.S.-based participants. Although AMT samples are more heterogeneous than student samples used in most laboratory experiments (Berinsky et al., 2012), they are not representative of the U.S. population. Therefore, these results may not extend to some sections of the U.S. population, let alone to other countries. Regarding the United States, we believe that this is not a major limitation, because previous research found that AMTurkers respond to accuracy prompts in a similar fashion as participants recruited on Lucid, quota-sampled to match the American residents on age, gender, ethnicity, and geographical region (Pennycook et al., 2021); moreover, we reconducted our analyses by adding gender, age, and education, to check if they moderate the endorsing accuracy effect: We found very little and inconsistent evidence that this is the case (Tables S8-S10). However, it remains an open question whether our results hold in other countries. Third, our results say little about the duration of the effect. Table S11 reports the main analysis with a control for headlines display order, that we interpret as a proxy of time (Pennycook & Rand, 2022a; Roozenbeek et al., 2021a), its two-way interactions with the intervention dummies, and its three-way interactions with the intervention dummies and the headline veracity dummy. None of the two- and three-way interactions are statistically significant, suggesting that, within our experiment, the effects of the intervention do not significantly decay over time, not even when we consider headline veracity, at least when we consider linear decays; Figures S4 and S5 also found no evidence of non-linear decays. The lack of decay over time within our experiments is consistent with a similar result obtained by Pennycook and Rand (2022a) in their meta-analysis. Fourth, some of the headlines in our experiments contradict people’s lived experience (e.g., the existence of pink cows), thus raising the possibility that the accuracy endorsement intervention simply makes people pay more attention to headlines that contradict their lived experience. Table S12 rules out this possibility by excluding from the analyses headlines that contradict lived experience; the main results remain qualitatively the same. Fifth, we defined overall engagement as likes + shares. Although this represents an advance compared with previous work, which considered only sharing, it is still limited as it does not consider other forms of engagement, such as non-news sharing, or engagement with posts without links. Related to this, the null effect on overall sharing and engagement that we found descends from the fact that, in our experiments, half of news were real, and half were fake. In reality, the percentage of fake news consumed by social media users is a small percentage of the total news (Allen et al., 2020). Therefore, if our effects translate into actual social media behavior and if the endorsing accuracy intervention does not create any “collateral damage” on other types of content, then it is likely that it would increase overall sharing and engagement. Testing the effectiveness of endorsing accuracy on total engagement in an environment that more closely resembles a social media in terms of content distribution is an important direction for future work. Sixth, the endorsing accuracy intervention only allows users to signal the fact that they are sharing because they think that a headline is accurate. In view of applications, it would be important to test variants of this intervention that allows people to signal also other motivations for sharing (e.g., “I think this news is amusing”). Seventh, we did not test for carry-over effects; that is, does our treatment still have an effect if it is not reminded in every post? Although we deliberately reminded the treatment in every post because we wanted to simulate a structural change in a social media, we think that studying carry-over effects would be of theoretical interest.

Despite these limitations, our results show that endorsing accuracy may decrease fake news sharing and increase real news sharing. Therefore, endorsing accuracy could be a promising intervention for increasing the quality of information on social media.

## Supplemental Material

sj-docx-1-psp-10.1177_01461672221117691 – Supplemental material for “I Think This News Is Accurate”: Endorsing Accuracy Decreases the Sharing of Fake News and Increases the Sharing of Real News Click here for additional data file. Supplemental material, sj-docx-1-psp-10.1177_01461672221117691 for “I Think This News Is Accurate”: Endorsing Accuracy Decreases the Sharing of Fake News and Increases the Sharing of Real News by Valerio Capraro and Tatiana Celadin in Personality and Social Psychology Bulletin

## Notes

1 We had preregistered the use of mixed-effects linear regression. However, here we report the output of linear regression with a two-dimensional clustering, to align our work with the most recent works on the topic (e.g., Pennycook et al., 2021). In any case, all the main results of this article remain qualitatively similar if we use a mixed-effects linear regression with robust standard errors clustered at the participant level. Similarly, we obtain qualitatively similar results if, instead of a linear model, we fit a logistic model. For ease of interpretation of the coefficients, in the text, we report the output of the linear model.

2 Note that this assumption is quite strong, because it is based on the underlying assumption that all participants pay attention to the accuracy nudges; however, we make this assumption for simplicity: In reality, our argument holds under a much weaker and more realistic assumption, that is, that the proportion of participants not paying attention to the nudges does not significantly vary across experimental treatments—an assumption that is more realistic because, given the similarities across treatments, there is no reason to suspect that participants were more attentive in one treatment than in the other.

3 We did not report the comparison with the baseline, because in the baseline we cannot assume that *U*(*x*) *= b*_1_(−*F*(*x*)), since accuracy is not salient. For the same reason, we cannot even make the weaker assumption that the proportion of participants for which accuracy is not salient is the same as in the experimental treatments, where accuracy is salient. In any case, for completeness, we estimated θ and μ also in the baseline and found that, in line with our interpretation, they are even further away from endorsing accuracy than accuracy salience/fake alert.
