# Supplementary material for: “I Think This News Is Accurate”: Endorsing Accuracy Decreases the Sharing of Fake News and Increases the Sharing of Real News
Source: Pers Soc Psychol Bull. 2022 Aug 21;49(12):1635–45. doi: 10.1177/01461672221117691 (PMC10637098; doi:10.1177/01461672221117691)
Supplement: sj-docx-1-psp-10.1177_01461672221117691 – Supplemental material for “I Think This News Is Accurate”: Endorsing Accuracy Decreases the Sharing of Fake News and Increases the Sharing of Real News [file sj-docx-1-psp-10.1177_01461672221117691.docx]

**Supplemental Material**

for

**“I think this news is accurate”: Endorsing accuracy decreases the sharing of fake news, increases the sharing of real news, and keeps overall engagement constant**

Table of contents

Detailed analysis……………………………………………………………...2

Additional analysis…………………………………………………………...20

Supplementary references…………………………………………………….30

**Detailed analysis**

**Study 1**

We pre-registered a sample size of N=600. We eliminated 4 multiple IP addresses and multiple Turk IDs. Then, as pre-registered, we eliminated 43 participants because they reported that they do not use or share content on social media. In doing so, we remain with 550 participants (198 in the *baseline*, 198 in the *accuracy endorsement* condition, and 197 in the *fake alert* condition). All the participants responded correctly to the control question. Therefore, we have only one secondary analysis, corresponding to eliminating participants who indicated having searched online for the headlines during the study (*baseline*: N = 16; *accuracy endorsement*: N= 15; *fake alert*: N= 14). The regression results are reported in Table S1. Model 1a reports the primary analysis, Model 2a the secondary analysis. Models 1b and Model 2b report an exploratory, non-pre registered analysis, controlling for type of news (political, COVID-19, others). The details of the primary analysis were already reported in the main text. Below, we discuss the details of the secondary analysis.

A linear regression with robust standard errors clustered on participants and headlines reveals that the interaction between headline veracity (real vs fake) and the *accuracy endorsement* condition is significant while the interaction between headline veracity and the *fake alert* condition is not significant (*real news X fake alert*: b = 0.029, p = 0.377, t = 0.88, 95% CI [-0.035; 0.093]; *real news X accuracy endorsement*: b = 0.236, p < 0.001, z=5.68, 95% CI [0.154; 0.317]). The coefficients of the two interactions (*real news X fake alert* vs. *real news X accuracy endorsement*) are statistically different, F(1, 12114) = 31.58, p < 0.001. Post-hoc analyses show that there is no difference in the sharing intentions of fake news when we compare the *baseline* and the *fake alert* conditions, b = -0.008, p = 0.767. By contrast, the *accuracy endorsement* condition decreases the sharing intentions of fake news with respect to both the *baseline* and the *fake alert* condition (*baseline* vs *accuracy endorsement*: b = -0.055; p = 0.034; *fake alert* vs *accuracy endorsement:* b = -0.047, p = 0.038). Regarding real news, the *accuracy endorsement* condition increases the sharing intentions of real news with respect to both the *baseline* and the *fake alert* conditions (*baseline* vs *accuracy endorsement*: b = 0.181; p < 0.001; *fake alert* vs *accuracy endorsement*: b = 0.160, p < 0.001), whereas there is no difference between the sharing intention of real news in the *baseline* and the *fake alert* conditions, b = 0.021, p = 0.479. Overall (real and fake news together), the *accuracy endorsement* condition slightly increases the sharing intentions with respect to both the *baseline* and the *fake alert* condition (*baseline* vs *accuracy endorsement*: b = 0.063, p = 0.066; *fake alert* vs *accuracy endorsement*: b = -0.057, p = 0.068), while there is no difference between the *baseline* and the *fake alert* conditions in the sharing intentions, b = 0.007, p = 0.778.

Models 1b and 2b report linear regressions with robust standard errors clustered at the participant level and show that the main results are robust to including controls on type of news (political, COVID-19, other) and all the relevant interactions. We note that the significant three-way interactions Politics X Endorsement X Real and (to a lesser extent) COVID X Endorsement X Real suggest that the effect of endorsing accuracy is particularly strong on real political or COVID-related headlines. However, these results were not replicated in any of the subsequent studies, therefore we think they might be a false positive.

| **Sharing** | **Model 1a** | **Model 1b** | **Model 2a** | **Model 2b** |
| --- | --- | --- | --- | --- |
| Fake alert | -0.028  (0.029) | -0.025  (0.028) | -0.008  (0.027) | -0.005  (0.026) |
| Accuracy endorsement | -0.077***  (0.028) | -0.070***  (0.027) | -0.055**  (0.026) | -0.050**  (0.024) |
| Real | 0.205***  (0.037) | 0.214***  (0.023) | 0.220***  (0.039) | 0.229***  (0.024) |
| Alert x Real | 0.026  (0.031) | 0.010  (0.033) | 0.029  (0.033) | 0.014  (0.034) |
| Endorsement x Real | 0.239***  (0.040) | 0.203***  (0.035) | 0.236***  (0.042) | 0.201***  (0.036) |
| Politics |  | 0.007  (0.025) |  | 0.008  (0.026) |
| Politics x Real |  | -0.116***  (0.037) |  | -0.134***  (0.039) |
| Politics x Alert |  | -0.017  (0.036) |  | -0.007  (0.037) |
| Politics x Endorsement |  | -0.039  (0.032) |  | -0.034  (0.033) |
| Politics x Real x Alert |  | 0.101*  (0.056) |  | 0.099*  (0.058) |
| Politics x Real x Endorsement |  | 0.270***  (0.053) |  | 0.284***  (0.054) |
| Covid |  | 0.032*  (0.018) |  | 0.029  (0.019) |
| Covid x Real |  | 0.004  (0.028) |  | 0.012  (0.029) |
| Covid x Alert |  | -0.011  (0.026) |  | -0.016  (0.026) |
| Covid x Endorsement |  | -0.020  (0.024) |  | -0.014  (0.024) |
| Covid x Real x Alert |  | 0.045  (0.038) |  | 0.040  (0.039) |
| Covid x Real x Endorsement |  | 0.082**  (0.039) |  | 0.067*  (0.040) |
| Constant | 0.211***  (0.022) | 0.205***  (0.020) | 0.164***  (0.020) | 0.159***  (0.018) |
| Excluding Searched Online | No | No | Yes | Yes |
| Observations | 13200 | 13200 | 12120 | 12120 |

**Table S1.** *Study 1. Linear regressions with robust standard errors clustered on* *participants and headlines (Model 1a and 2a) or on participants only (Model 1b and 2b), predicting sharing intentions. Model 1 refers to the primary analysis, Model 2 to the secondary analysis. Robust standard errors (clustered) in brackets. Significance levels: *: p < 0.1, **: p < 0.05, ***: p < 0.01.*

**Study 2**

We pre-registered a sample size of N=600. We eliminated 4 multiple IP addresses and Turk IDs. Then, as pre-registered, we eliminated 47 participants because they reported that they do not use or share content on social media. In doing so, we remain with 558 participants (197 in the *baseline*, 175 in the *accuracy endorsement* condition, and 186 in the *fake alert* condition). All the participants responded correctly to the control question. Therefore, we have only one secondary analysis, corresponding to eliminating participants who indicated having searched online for the headlines during the study (*baseline*: N = 19; *accuracy endorsement*: N= 15; *fake alert*: N= 21). Regression results are reported in Table S2. Model 1a reports the primary analysis, Model 2a the secondary analysis. Models 1b and 2b add controls on type of news (politics, COVID-19, others). The details of the primary analysis were already reported in the main text. Below, we discuss the details of the secondary analysis.

A linear regression with robust standard errors clustered on participants and headlines reveals that the interactions between headline veracity (*real* vs *fake*) and condition are both significant (*real news X fake alert*: b = 0.056, p = 0.019, t = 2.35, 95% CI [0.09; 0.102]; *real news X accuracy endorsement*: b = 0.175, p < 0.001, t = 6.28; 95% CI [0.121; 0.230]). The coefficients of the two interactions (*real news X fake alert* vs. *real news X accuracy endorsement*) are statistically different, F(1, 12066) = 20.72, p < 0.001. Post-hoc analyses show that the sharing intentions of fake news in the *fake alert* condition is lower than in the *baseline*, b = -0.053, p = 0.044. The sharing intentions of fake news in the *accuracy endorsement* are lower than they are in both the *baseline*, b = -0.108; p < 0.001, and the *fake alert* condition, b = -0.055, p = 0.010. The sharing intentions of real news in the *accuracy endorsement* condition are higher than they are in both the *baseline*, b = 0.068; p = 0.023, and the *fake* condition, b = 0.065, p = 0.030. There is no difference between the sharing intentions of real news in the *baseline* and the *fake alert* condition, b = 0.003, p = 0.915. Overall (fake and real news together), neither the *accuracy endorsement* nor the *fake alert* significantly decreases the sharing intentions with respect to the *baseline* (*baseline* vs *accuracy endorsement*: b = -0.020, p = 0.496; *baseline* vs *fake alert*: b = -0.025, p = 0.306). Similarly, there is no difference in the overall sharing intentions between the *accuracy endorsement* and the *fake alert* condition, b = 0.005, p = 0.849.

Models 1b and 2b report linear regressions with robust standard errors clustered at the participant level and show that the main results are robust to including controls on type of news (political, COVID-19, other) and the relevant interactions.

| **Sharing** | **Model 1a** | **Model 1b** | **Model 2a** | **Model 2b** |
| --- | --- | --- | --- | --- |
| Fake alert | -0.051**  (0.025) | -0.046*  (0.027) | -0.053**  (0.027) | -0.047*  (0.028) |
| Accuracy endorsement | -0.089***  (0.024) | -0.093***  (0.025) | -0.108***  (0.024) | -0.109***  (0.026) |
| Real | 0.042*  (0.024) | 0.036**  (0.018) | 0.047*  (0.026) | 0.044**  (0.019) |
| Alert x Real | 0.050**  (0.021) | 0.053**  (0.024) | 0.056**  (0.024) | 0.056**  (0.026) |
| Endorsement x Real | 0.156***  (0.025) | 0.154***  (0.027) | 0.175***  (0.028) | 0.171***  (0.029) |
| Politics |  | -0.041*  (0.024) |  | -0.051**  (0.024) |
| Politics x Real |  | -0.010  (0.032) |  | -0.016  (0.034) |
| Politics x Alert |  | 0.014  (0.033) |  | 0.018  (0.032) |
| Politics x Endorsement |  | 0.029  (0.031) |  | 0.029  (0.030) |
| Politics x Real x Alert |  | -0.094**  (0.045) |  | -0.090*  (0.046) |
| Politics x Real x Endorsement |  | -0.042  (0.047) |  | -0.049  (0.047) |
| Covid |  | 0.018  (0.017) |  | 0.023  (0.018) |
| Covid x Real |  | 0.041  (0.027) |  | 0.029  (0.028) |
| Covid x Alert |  | -0.034  (0.024) |  | -0.043*  (0.024) |
| Covid x Endorsement |  | 0.005  (0.023) |  | -0.005  (0.023) |
| Covid x Real x Alert |  | 0.030  (0.039) |  | 0.044  (0.040) |
| Covid x Real x Endorsement |  | 0.036  (0.040) |  | 0.052  (0.042) |
| Constant | 0.218***  (0.020) | 0.218***  (0.020) | 0.202***  (0.021) | 0.202***  (0.021) |
| Excluding Searched Online | No | No | Yes | Yes |
| Observations | 13392 | 13392 | 12072 | 12072 |

**Table S2.** *Study 2. Linear regressions with robust standard errors clustered on* *participants and headlines (Model 1a and 2a) or on participants only (Model 1b and 2b), predicting sharing intentions. Model 1 refers to the primary analysis, Model 2 to the secondary analysis. Robust standard errors (clustered) in brackets. Significance levels: *: p < 0.1, **: p < 0.05, ***: p < 0.01.*

As pre-registered we run all the analyses for the *liking intention*, *l(p,h*), defined for every participant *p* and headline *h*, and representing *p*’s decision of whether to like *h* or not. Fig. S1 reports the average *l(p,h)* split by headline veracity (*real* vs *fake*) and condition (*baseline* vs *fake alert* vs *accuracy endorsement*).


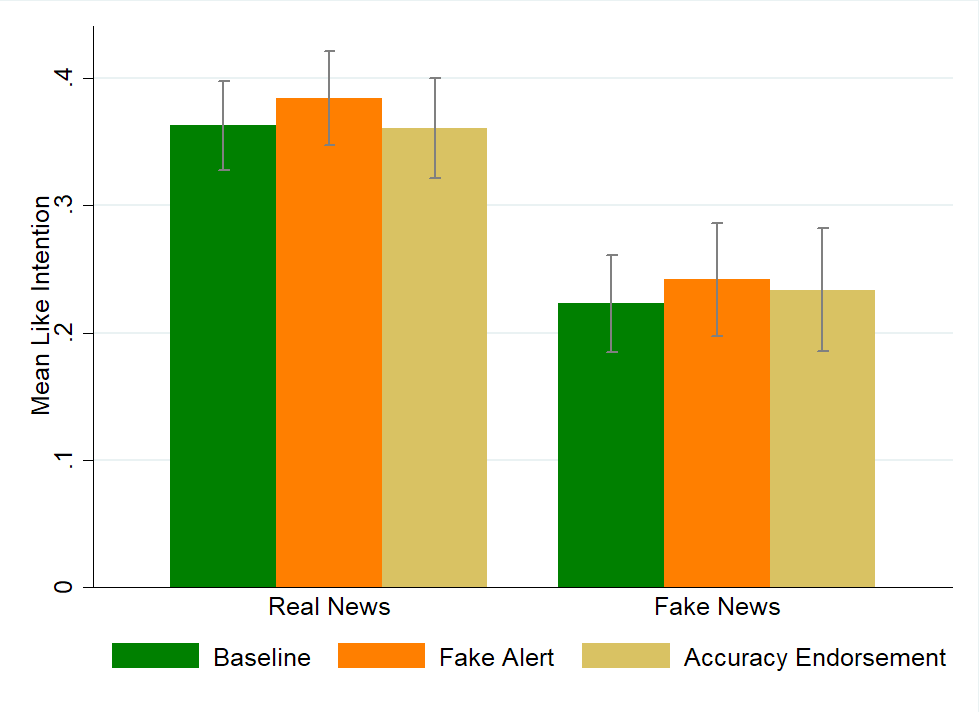


**Fig. S1**. Average liking intentions in Study 2 split by headline veracity (*real* vs *fake*) and condition (*baseline* vs *fake alert* vs *accuracy endorsement*). Error bars represent 95% CI clustered at the participant level.

Table S3, Model 1, reports the primary analysis. The only statistically significant effect that we observe is that of Real; participants tend to like real news more than fake news. The coefficients of the two interactions (*real news X fake alert* vs. *real news X accuracy endorsement*) are also not statistically different, F(1, 13386) = 0.58, p = 0.445. Post-hoc analyses show that there is no difference in the liking intentions of fake news across treatments (*baseline* vs *fake alert*: b = 0.022; p = 0.420; *baseline* vs *accuracy endorsement*: b = -0.002, p = 0.938; *fake alert* vs *accuracy endorsement*: b = -0.024, p = 0.352). Similarly, for real news, there is no difference in the liking intention across treatments (*baseline* vs *fake alert*: b = 0.019; p = 0.513; *baseline* vs *accuracy endorsement*: b = 0.011, p = 0.717; *fake alert* vs *accuracy endorsement*: b = -0.008, p = 0.795). And similarly, there is no difference in the liking intention across treatments in the overall liking intentions (*baseline* vs *accuracy endorsement*: b = 0.005, p = 0.863; *fake alert* vs *accuracy endorsement*: b =-0.016, p = 0.552; *baseline* vs *fake alert* b = 0.020, p = 0.423).

As pre-registered, we also run the analysis for the liking intentions after eliminating participants who indicated having searched online for the headlines during the study**.** Table S3, Model 2, reports this secondary analysis. Again, the only statistically significant effect is that of Real. The coefficients of the two interactions (*real news X fake alert* vs. *real news X accuracy endorsement*) are also not statistically different, F(1, 12066) = 0.73, p = 0.393. Post-hoc analyses show that there is no difference in the liking intentions for fake news across treatments (*baseline* vs *fake alert*: b = 0.013, p = 0.627; *baseline* vs *accuracy endorsement*: b = -0.017, p = 0.494; *fake alert* vs *accuracy endorsement*: b = -0.030, p = 0.203). Regarding real news, there is no difference in the liking intentions across treatments (*baseline* vs *fake alert*: b = 0.007; p = 0.790; *baseline* vs *accuracy endorsement*: b = -0.005, p = 0.873; *fake alert* vs *accuracy endorsement*: b = -0.012, p = 0.685). Overall (fake and real news together), there is no difference in the liking intentions across treatments (*fake alert* vs *accuracy endorsement*: b = -0.021, p = 0.399; *baseline* vs *fake alert*: b = 0.010, p = 0.677; *baseline* vs *accuracy endorsement*: b = -0.011, p = 0.661).

| **Liking** | **Model 1** | **Model 2** |
| --- | --- | --- |
| Fake alert | 0.019  (0.029) | 0.007  (0.027) |
| Accuracy endorsement | 0.011  (0.030) | -0.005  (0.030) |
| Real | 0.140***  (0.050) | 0.149***  (0.054) |
| Alert x Real | 0.003  (0.023) | 0.005  (0.023) |
| Endorsement x Real | -0.013  (0.021) | -0.012  (0.023) |
| Constant | 0.223***  (0.023) | 0.191***  (0.024) |
| Search Online | Yes | No |
| Observations | 13392 | 12072 |

**Table S3.** *Study 2. Linear regression with robust standard errors clustered on* *participants and headlines, predicting liking intentions. Model 1 refers to the primary analysis, Model 2 to the secondary analysis. Robust standard errors clustered on participants and headlines in brackets. Significance levels: *: p < 0.1, **: p < 0.05, ***: p < 0.01.*

**Study 3**

We pre-registered a sample size of N=600. We eliminated 2 multiple IP addresses and Turk IDs. Then, as pre-registered, we eliminated 48 participants because they reported that they do not use or share content on social media. In doing so, we remain with 550 participants (186 in the *baseline*, 180 in the *accuracy endorsement* condition, and 184 in the *accuracy salience* condition). All the participants responded correctly to the control question. Therefore, we have only one secondary analysis, corresponding to eliminating participants who indicated having searched online for the headlines during the study (*baseline* N = 24; *accuracy endorsement*: N= 18; *accuracy salient*: N= 26). Regression results are reported in Table S4. Model 1a reports the primary analysis, Model 2a the secondary analysis. Models 1b and 2b add controls on type of news (political, COVID-19, others) and all the relevant interactions. The details of the primary analysis were already reported in the main text. Below, we discuss the details of the secondary analysis.

A linear regression with robust standard errors clustered on participants and headlines reveals that the interactions between headline veracity and condition are both significant (*real news X accuracy salience*: b = 0.102, p = 0.001, t = 3.22, 95% CI [0.040; 0.164]; *real news X accuracy endorsement*: b = 0.198, p < 0.001, t =5.84, 95% CI [0.131; 0.264]). The coefficients of the two interactions (*real news X accuracy salience* vs. *real news X accuracy endorsement*) are statistically different, F(1, 11562) = 10.26, p = 0.001. Post-hoc analyses show that the sharing intentions of fake news in the *accuracy salience* condition is lower than in the *baseline*, b = -0.073, p = 0.007. The *accuracy endorsement* condition decreases the sharing intentions of fake news headlines with respect to the *baseline* but not with respect to the *accuracy salience* condition (*baseline* vs *accuracy endorsement*: b = -0.111; p < 0.001; *accuracy salience* vs. *accuracy endorsement*: b = -0.038, p = 0.110). Regarding real news, *accuracy endorsement* increases the sharing intentions of real news with respect to both the *baseline* and the *accuracy salience* condition (*baseline* vs *accuracy endorsement*: b = 0.086 p = 0.014; *accuracy salience* vs. *accuracy endorsement*: b = 0.058, p = 0.044). The *accuracy salience* condition does not increase the sharing intentions of real news with respect to the *baseline*, b = 0.029, p = 0.393. Overall (fake and real news together), there is no difference in the sharing intentions across conditions (*baseline* vs *accuracy endorsement*: b = -0.012, p = 0.705; *accuracy endorsement* vs *accuracy salient*: b = 0.010, p = 0.674; *accuracy salient* vs *baseline*: b = -0.022, p = 0.427).

Models 1b and 2b show that the main results are robust to including controls on type of news and all the relevant interactions.

| **Sharing** | **Model 1a** | **Model 1b** | **Model 2a** | **Model 2b** |
| --- | --- | --- | --- | --- |
| Accuracy salience | -0.097***  (0.027) | -0.102***  (0.027) | -0.073***  (0.027) | -0.074***  (0.028) |
| Accuracy endorsement | -0.133***  (0.026) | -0.131***  (0.026) | -0.111***  (0.026) | -0.106***  (0.026) |
| Real | 0.059**  (0.027) | 0.059***  (0.020) | 0.075***  (0.026) | 0.076***  (0.021) |
| Salient x Real | 0.099***  (0.028) | 0.106***  (0.028) | 0.102***  (0.032) | 0.103***  (0.031) |
| Endorsement x Real | 0.196***  (0.032) | 0.189***  (0.030) | 0.198***  (0.034) | 0.187***  (0.032) |
| Politics |  | -0.010  (0.022) |  | -0.008  (0.021) |
| Politics x Real |  | -0.070**  (0.034) |  | -0.070**  (0.034) |
| Politics x Salience |  | 0.050  (0.033) |  | 0.029  (0.031) |
| Politics x Endorsement |  | 0.023  (0.031) |  | 0.001  (0.030) |
| Politics x Real x Salience |  | -0.057  (0.051) |  | -0.040  (0.052) |
| Politics x Real x Endorsement |  | -0.012  (0.050) |  | -0.002  (0.052) |
| Covid |  | 0.023  (0.018) |  | 0.023  (0.019) |
| Covid x Real |  | 0.032  (0.024) |  | 0.026  (0.025) |
| Covid x Salience |  | 0.002  (0.024) |  | -0.009  (0.025) |
| Covid x Endorsement |  | -0.024  (0.024) |  | -0.033  (0.024) |
| Covid x Real x Salience |  | -0.010  (0.038) |  | 0.013  (0.040) |
| Covid x Real x Endorsement |  | 0.047  (0.036) |  | 0.063*  (0.038) |
| Constant | 0.249***  (0.024) | 0.246***  (0.021) | 0.209***  (0.023) | 0.206***  (0.021) |
| Excluding Searched Online | No | No | Yes | Yes |
| Observations | 13200 | 13200 | 11568 | 11568 |

**Table S4.** *Study 3. Linear regressions with robust standard errors clustered on* *participants and headlines (Model 1a and 2a) or on participants only (Model 1b and 2b), predicting sharing intentions. Model 1 refers to the primary analysis, Model 2 to the secondary analysis. Robust standard errors clustered at the participant level in brackets. Significance levels: *: p < 0.1, **: p < 0.05, ***: p < 0.01.*

As pre-registered we run all the analysis for the liking intentions. Fig. S2 reports the average liking intentions split by headline veracity (*real* vs *fake*) and condition (*baseline* vs *accuracy salient* vs *accuracy endorsement*).


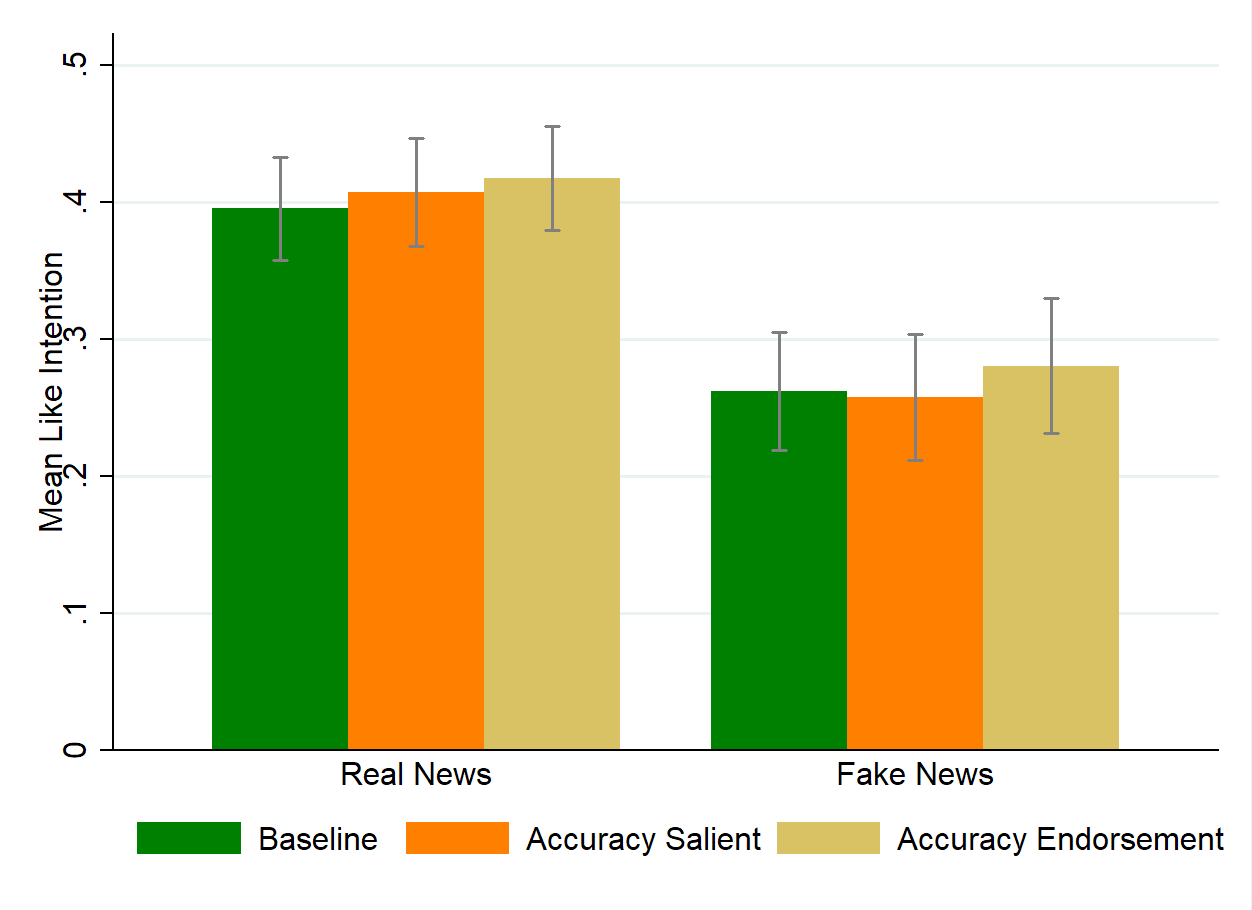


**Fig. S2**. Average liking intentions in Study 3 split by headline veracity (*real* vs *fake*) and condition (*baseline* vs *accuracy salient* vs *accuracy endorsement*). Error bars represent 95% CI clustered at the participant level.

Table S5, Model 1, reports the primary analysis. The only statistically significant effect is that of Real: participants tend to like real news more than they like fake news. The coefficients of the two interactions (*real news X accuracy salience* vs. *real news X accuracy endorsement*) are not statistically different, F(1, 13010) = 0.22, p = 0.641. Post-hoc analyses show that there is no difference in the liking intentions of fake news across conditions (*baseline* vs *accuracy salience*: b = 0.019; p = 0.577; *baseline* vs *accuracy endorsement*: b = -0.005, p = 0.883; *accuracy endorsement* vs *accuracy salience:* b = 0.023, p = 0.475). Similarly, there is no difference in the liking intentions of real news across treatments (*baseline* vs *accuracy salient*: b = 0.012; p = 0.664; *baseline* vs *accuracy endorsement*: b =0.022, p = 0.420; *accuracy salience* vs *accuracy endorsement*: b = 0.010, p = 0.739). Similarly, there is no difference across treatments in the overall liking intentions (*baseline* vs *accuracy salient*: b = 0.007, p = 0.798; *accuracy salience* vs *accuracy endorsement*: b = 0.013, p = 0.633; *baseline* vs *accuracy endorsement:* b = 0.020, p = 0.454).

As pre-registered, we also run the analysis for the liking intentions by eliminating participants who indicated having searched online for the headlines during the study**.** Table S5, Model 2, reports this secondary analysis. Again, the only significant effect is that of Real. The coefficients of the two interactions (*real news X accuracy salient* vs. *real news X accuracy endorsement*) are not statistically different, F(1, 11404) = 0.31, p = 0.578. Post-hoc analyses show that there is no difference in the liking intentions of fake news across conditions (*baseline* vs *accuracy salience*: b = -0.005, p = 0.858; *baseline* vs *accuracy endorsement*: b = 0.040, p = 0.220; *accuracy salience* vs *accuracy endorsement*: b = 0.045, p = 0.164). Similarly, there is no difference across conditions in the liking intentions of real news (*baseline* vs *accuracy salience*: b = 0.016, p = 0.545; *baseline* vs *accuracy endorsement*: b = 0.043, p = 0.147; *accuracy salience* vs *accuracy endorsement*: b = 0.027, p = 0.395). And, similarly, there is no difference across conditions in the overall liking intentions (*baseline* vs *accuracy endorsement*: b = 0.041, p = 0.123; *accuracy salience* vs *accuracy endorsement*: b = 0.032, p = 0.239; *baseline* vs *accuracy salience* conditions: b = 0.009, p = 0.723).

| **Liking** | **Model 1** | **Model 2** |
| --- | --- | --- |
| Accuracy salient | -0.005  (0.031) | -0.005  (0.030) |
| Accuracy endorsement | 0.019  (0.033) | -0.040  (0.032) |
| Real | 0.133**  (0.053) | 0.146***  (0.057) |
| Salience x Real | 0.017  (0.024) | 0.021  (0.025) |
| Endorsement x Real | 0.004  (0.027) | 0.003  (0.030) |
| Constant | 0.262***  (0.027) | 0.212***  (0.026) |
| Search Online | Yes | No |
| Observations | 13016 | 11410 |

**Table S5.** *Study 3. Linear regressions with robust standard errors clustered on* *participants and headlines, predicting liking intentions. Model 1 refers to the primary analysis, Model 2 to the secondary analysis. Robust standard errors (clustered) in brackets. Significance levels: *: p < 0.1, **: p < 0.05, ***: p < 0.01.*

**Study 4**

We pre-registered a sample size of N=400. We eliminated 3 multiple IP addresses and 3 Turk IDs. Then, as pre-registered, we eliminated 28 participants because they reported that they do not use or share content on social media. In doing so, we remain with 372 participants (186 in the *baseline*, and 186 in the *accuracy endorsement* condition). All the participants responded correctly to the control question. Therefore, we have only one secondary analysis, corresponding to eliminating participants who indicated having searched online for the headlines during the study (*baseline* N = 29; *accuracy endorsement*: N= 32). Regression results are reported in Table S6. Model 1a reports the primary analysis, Model 2a the secondary analysis. Models 1b and 2b add controls on type of news (political, COVID-19, others) and all the relevant interactions. The details of the primary analysis were already reported in the main text. Below, we discuss the details of the secondary analysis.

A linear regression with robust standard errors clustered on participants and headlines reveals that the interactions between headline veracity and condition is significant (*real news X accuracy endorsement*: b = 0.144, p < 0.001, t = 5.43, 95% CI [0.091; 0.196]). The sharing of fake news is lower in the *accuracy endorsement* condition than it is in the *baseline* (b = -0.085; p = 0.002). By contrast, the sharing of real news is higher in the *accuracy endorsement* than it is in the *baseline* (b = 0.059; p = 0.054). Overall (fake and real news together), there is no significant difference in the sharing intentions between the *accuracy endorsement* condition and the *baseline* (b = -0.013, p = 0.699).

Models 1b and 2b show that the main results are robust to including controls on type of news (political, covid-related, others) and all the relevant interactions.

| **Sharing** | **Model 1a** | **Model 1b** | **Model 2a** | **Model 2b** |
| --- | --- | --- | --- | --- |
| Accuracy endorsement | -0.071***  (0.027) | -0.081***  (0.029) | -0.085***  (0.028) | -0.093***  (0.032) |
| Real | 0.037  (0.052) | 0.061**  (0.024) | 0.037  (0.052) | 0.076***  (0.026) |
| Endorsement x Real | 0.126***  (0.025) | 0.143***  (0.035) | 0.144***  (0.027) | 0.142***  (0.039) |
| Politics |  | 0.034  (0.031) |  | 0.040  (0.032) |
| Politics x Real |  | -0.066  (0.042) |  | -0.121***  (0.043) |
| Politics x Endorsement |  | -0.070*  (0.042) |  | 0.046  (0.044) |
| Politics x Real x Endorsement |  | -0.079  (0.061) |  | -0.000  (0.064) |
| Covid |  | 0.066**  (0.029) |  | 0.040  (0.028) |
| Covid x Real |  | -0.071*  (0.037) |  | -0.076**  (0.037) |
| Covid x Endorsement |  | -0.011  (0.040) |  | 0.001  (0.041) |
| Covid x Real x Endorsement |  | -0.009  (0.054) |  | 0.004  (0.056) |
| Constant | 0.284***  (0.029) | 0.268***  (0.023) | 0.260***  (0.027) | 0.247***  (0.025) |
| Excluding Searched Online | No | No | Yes | Yes |
| Observations | 4464 | 4464 | 3732 | 3732 |

**Table S6.** *Study 4. Linear regressions with robust standard errors clustered on* *participants and headlines (Model 1a and 2a) or on participants only (Model 1b and 2b), predicting sharing intentions. Model 1 refers to the primary analysis, Model 2 to the secondary analysis. Robust standard errors (clustered) in brackets. Significance levels: *: p < 0.1, **: p < 0.05, ***: p < 0.01.*

As pre-registered, we run all the analysis for the liking intention. Fig. S3 reports the average liking intentions split by headline veracity (*real* vs *fake*) and condition (*baseline* vs *accuracy endorsement*).


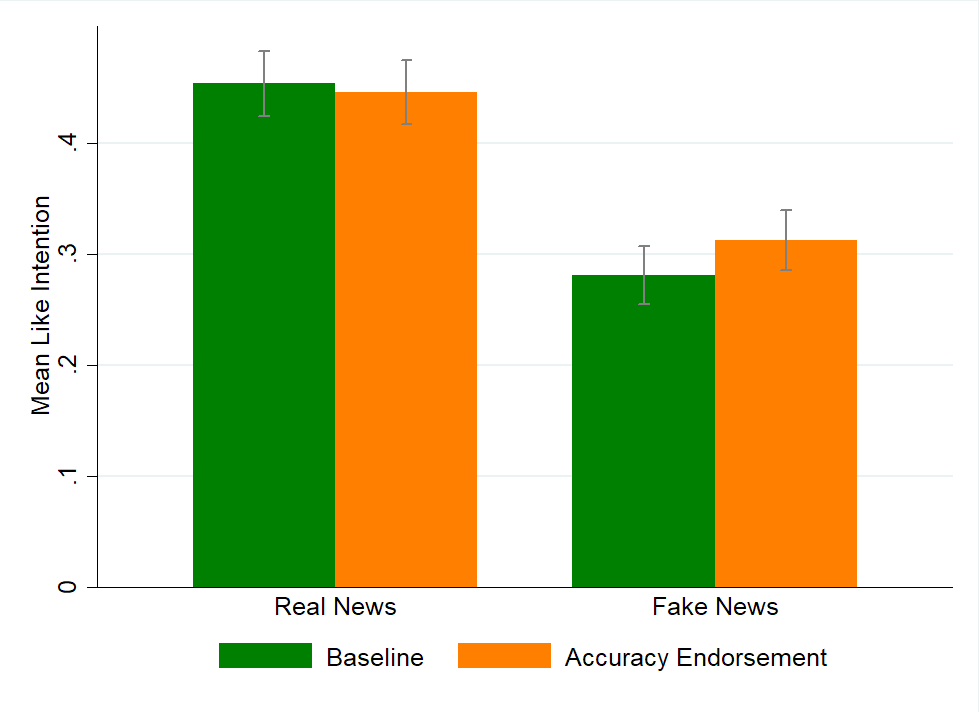


**Fig. S3**. Mean intentions to like news headlines in Study 4 split by headline veracity (*real* vs *fake*) and condition (*baseline* vs *accuracy salient* vs *accuracy endorsement*). Error bars represent 95% CI clustered at the participant level.

Table S7, Model 1, reports the primary analysis. The only statistically significant effect is that of Real: participants tend to like real news more than they like fake news. Post-hoc analyses show that there is no difference between conditions in the intentions to like fake news (b = 0.031, p = 0.383) nor there is in the intentions to like real news (b = -0.008, p = 0.764). Similarly, there is no difference in the overall liking intentions (b = 0.012, p = 0.678).

As pre-registered, we also run the analysis for the liking intentions after eliminating participants who indicated having searched online for the headlines during the study**.** Table S7, Model 2, reports the secondary analysis. Again, we find that participants tend to like real news more than fake news, as the main effect of *accuracy endorsement* is slightly significant. Post-hoc analyses show no difference between conditions in the intentions to like fake news (b = 0.036, p = 0.349) and in the intentions to like real news (b = -0.006, p = 0.814). Similarly, there is no difference in the overall intentions to like news headlines, b = 0.015, p = 0.611.

| **Liking** | **Model 1** | **Model 2** |
| --- | --- | --- |
| Accuracy endorsement | 0.031  (0.035) | 0.036  (0.037) |
| Real | 0.173***  (0.056) | 0.205***  (0.056) |
| Endorsement x Real | -0.039  (0.029) | -0.042  (0.031) |
| Constant | 0.281***  (0.048) | 0.227***  (0.048) |
| Search Online | Yes | No |
| Observations | 4464 | 3732 |

**Table S7**. *Study 4. Linear regressions with robust standard errors clustered on* *participants and headlines, predicting liking intentions. Model 1 refers to the primary analysis, Model 2 to the secondary analysis. Robust standard errors (clustered) in brackets. Significance levels: *: p < 0.1, **: p < 0.05, ***: p < 0.01.*

**Additional analysis**

**The moderating role of gender, education, and age**

To check if the effects of the interventions depend on gender, age, or education, in this subsection we report the main analyses for Studies 1-4, with additional controls for each of these variables and their two-way and three-way interactions with condition and headline veracity.

In Table S8, gender is a dummy variable that takes value 1 if the participant is female, 0 otherwise; we exclude those participants who indicated their gender as “other” or replied that they prefer not to tell (Study 1: N=4; Study 2: N=6; Study 3: N=1; Study 4: N=5). Table S8 shows that the two-way interactions Endorsement X Gender and the three-way interactions Endorsement X Real X Gender are never statistically significant, suggesting the effect of the accuracy endorsement intervention is similar for women and men (two-way interactions), even when we take into account headline veracity (three-way interactions). A similar result holds also for the other conditions (*fake aler*t and *accuracy salience*).

| **Sharing** | **Study 1** | **Study 2** | **Study 3** | **Study 4** |
| --- | --- | --- | --- | --- |
| Fake Alert | -0.002  (0.040) | -0.059*  (0.036) |  |  |
| Accuracy salience |  |  | -0.138***  (0.037) |  |
| Accuracy endorsement | -0.049  (0.038) | -0.117***  (0.030) | -0.181***  (0.038) | -0.079**  (0.035) |
| Real | 0.203***  (0.037) | 0.040*  (0.024) | 0.059**  (0.027) | 0.038  (0.052) |
| Alert x Real | -0.009  (0.040) | 0.039  (0.026) |  |  |
| Salience x Real |  |  | 0.106***  (0.031) |  |
| Endorsement x Real | 0.231***  (0.048) | 0.180***  (0.036) | 0.187***  (0.038) | 0.122***  (0.037) |
| Gender | 0.018  (0.035) | -0.045  (0.032) | -0.092**  (0.038) | -0.043  (0.039) |
| Alert x Gender | -0.052  (0.051) | 0.019  (0.048) |  |  |
| Salience x Gender |  |  | 0.079  (0.050) |  |
| Endorsement x Gender | -0.063  (0.047) | 0.055  (0.045) | 0.092*  (0.047) | 0.027  (0.049) |
| Alert x Real x Gender | 0.071  (0.044) | 0.025  (0.027) |  |  |
| Salience x Real x Gender |  |  | -0.012  (0.040) |  |
| Endorsement x Real x Gender | 0.020  (0.049) | -0.050  (0.040) | 0.018  (0.044) | 0.005  (0.045) |
| Constant | 0.203***  (0.028) | 0.240***  (0.026) | 0.297***  (0.033) | 0.299***  (0.037) |
| Observations | 13104 | 13248 | 13176 | 4428 |

**Table S8.** *Linear regression with robust standard errors clustered on participants and headlines for studies 1-4, where we control for gender and its two-way and three-way interactions with condition and headline veracity Robust standard errors (clustered) in brackets.*

Table S9 reports the main analysis for Studies 1-4 with additional controls on age and its two-way and three-way interactions with the condition and headline veracity. The two-way interaction Endorsement X Age is significant only in Study 4, while the three-way interaction Endorsement X Real X Age is significant in Studies 3-4. The other relevant two-way and three-way interactions, Alert X Age, Alert X Real X Age, Salience X Age, Salience X Real X Age, are never significant. This provides some evidence that the endorsing accuracy intervention, but not the other intervention, might interact with age. The evidence is nonetheless weak and inconsistent across studies. Future work might investigate this point in further detail.

| **Sharing** | **Study 1** | **Study 2** | **Study 3** | **Study 4** |
| --- | --- | --- | --- | --- |
| Fake Alert | 0.034  (0.087) | -0.097*  (0.052) |  |  |
| Accuracy salience |  |  | -0.065  (0.079) |  |
| Accuracy endorsement | -0.015  (0.074) | -0.062  (0.055) | -0.117  (0.074) | 0.119  (0.094) |
| Real | 0.205***  (0.037) | 0.042*  (0.024) | 0.059**  (0.027) | 0.037  (0.052) |
| Alert x Real | -0.024  (0.082) | -0.004  (0.051) |  |  |
| Salience x Real |  |  | 0.142**  (0.064) |  |
| Endorsement x Real | 0.165*  (0.085) | 0.131*  (0.067) | 0.035  (0.083) | -0.035  (0.072) |
| Age | 0.001  (0.002) | 0.001*  (0.000) | 0.000  (0.001) | 0.001  (0.002) |
| Alert x Age | -0.002  (0.002) | 0.001  (0.001) |  |  |
| Salience x Age |  |  | -0.001  (0.002) |  |
| Endorsement x Age | -0.002  (0.002) | -0.001  (0.001) | -0.000  (0.002) | -0.005**  (0.002) |
| Alert x Real x Age | 0.001  (0.002) | 0.001  (0.001) |  |  |
| Salience x Real x Age |  |  | -0.001  (0.001) |  |
| Endorsement x Real x Age | 0.002  (0.002) | 0.001  (0.002) | 0.004**  (0.002) | 0.004**  (0.002) |
| Constant | 0.178***  (0.066) | 0.195***  (0.025) | 0.248***  (0.061) | 0.248***  (0.076) |
| Observations | 13200 | 13392 | 13200 | 4464 |

**Table S9.** *Linear regression with robust standard errors clustered on participants and headlines for studies 1-4, where we control for age and its two-way and three-way interactions with condition and headline veracity. Robust standard errors (clustered) in brackets. Significance levels: *: p < 0.1, **: p < 0.05, ***: p < 0.01.*

Finally, we study whether the effects of the interventions depend on the level of education. For each level of education, we use a dummy variable. Specifically, we have the following levels of education: “Less than a High School”, “High School Diploma”, “Vocational Training”, “Attended College”, “Bachelor's Degree”, “Graduate Degree”. Notice that we excluded from the analyses those participants with a level of education = “Less than High School” due to the fact that we have too few observations in this category (Study 1: N= 2; Study 2: N=1; Study 3: N=5; Study 4: N=4), which gives rise to some collinearities (due to the fact that there are no observations in this category in some treatments). Therefore, “high school diploma” is treated as the reference category. In Table S10, VC stands for “vocation training”, AC for “attended college”, and so forth. Also in this case, we find no consistent evidence that education has an effect. There are a few significant results, but they appear to be quite inconsistent; moreover, given the number of tests, virtually none of them survive to a correction for multiple comparisons.

| **Sharing** | **Study 1** | **Study 2** | **Study 3** | **Study 4** |
| --- | --- | --- | --- | --- |
| Fake Alert | 0.081  (0.072) | 0.007  (0.072) |  |  |
| Accuracy salience |  |  | -0.014  (0.083) |  |
| Accuracy endorsement | -0.064  (0.041) | -0.025  (0.068) | -0.201***  (0.056) | -0.285***  (0.087) |
| Real | 0.202***  (0.037) | 0.042*  (0.024) | 0.058**  (0.028) | 0.037  (0.051) |
| Alert x Real | 0.043  (0.060) | 0.042  (0.042) |  |  |
| Salience x Real |  |  | 0.040  (0.057) |  |
| Endorsement x Real | 0.263***  (0.072) | 0.047  (0.047) | 0.206***  (0.055) | 0.171***  (0.056) |
| Education VT | -0.048  (0.074) | 0.208**  (0.106) | -0.096  (0.164) | -0.107  (0.221) |
| Education AC | 0.021  (0.037) | 0.011  (0.059) | -0.022  (0.067) | -0.140  (0.088) |
| Education BD | 0.090**  (0.043) | 0.054  (0.055) | -0.015  (0.056) | -0.100  (0.086) |
| Education GD | 0.179***  (0.059) | 0.060  (0.063) | -0.061  (0.060) | -0.169*  (0.093) |
| Alert x Edu VT | 0.121  (0.127) | -0.231*  (0.131) |  |  |
| Salience x Edu VT |  |  | 0.001  (0.191) |  |
| Endorsement x Edu VT | -0.006  (0.083) | -0.290**  (0.119) | 0.084  (0.172) | 0.054  (0.230) |
| Alert x Real x Edu VT | -0.026  (0.124) | 0.048  (0.055) |  |  |
| Salience x Real x Edu VT |  |  | 0.013  (0.145) |  |
| Endorsement x Real x Edu VT | 0.056  (0.152) | 0.265***  (0.057) | 0.080  (0.131) | 0.236**  (0.104) |
| Alert x Edu AC | -0.124  (0.077) | -0.063  (0.085) |  |  |
| Salience x Edu AC |  |  | -0.189**  (0.096) |  |
| Endorsement x Edu AC | -0.016  (0.049) | -0.070  (0.080) | 0.034  (0.077) | 0.121  (0.101) |
| Alert x Real x Edu AC | -0.001  (0.071) | 0.000  (0.047) |  |  |
| Salience x Real x Edu AC |  |  | 0.221***  (0.069) |  |
| Endorsement x Real x Edu AC | -0.023  (0.081) | 0.078  (0.069) | 0.051  (0.067) | 0.059  (0.070) |
| Alert x Edu BD | -0.133*  (0.081) | -0.062  (0.080) |  |  |
| Salience x Edu BD |  |  | -0.079  (0.091) |  |
| Endorsement x Edu BD | 0.035  (0.060) | -0.069  (0.074) | 0.067  (0.065) | 0.286***  (0.094) |
| Alert x Real x Edu BD | -0.013  (0.067) | 0.026  (0.046) |  |  |
| Salience x Real x Edu BD |  |  | 0.033  (0.060) |  |
| Endorsement x Real x Edu BD | -0.042  (0.079) | 0.118**  (0.051) | -0.018  (0.058) | -0.105  (0.065) |
| Alert x Edu GD | -0.167*  (0.101) | -0.026  (0.095) |  |  |
| Salience x Edu GD |  |  | -0.060  (0.093) |  |
| Endorsement x Edu GD | -0.168**  (0.075) | -0.035  (0.085) | 0.146*  (0.076) | 0.260**  (0.110) |
| Alert x Real x Edu GD | -0.034  (0.074) | -0.047  (0.048) |  |  |
| Salience x Real x Edu GD |  |  | 0.018  (0.058) |  |
| Endorsement x Real x Edu GD | 0.013  (0.085) | 0.144**  (0.065) | -0.080  (0.070) | -0.058  (0.079) |
| Constant | 0.138***  (0.033) | 0.170***  (0.051) | 0.275***  (0.052) | 0.394***  (0.080) |
| Observations | 13152 | 13368 | 13080 | 4416 |

**Table S10.** *Linear regression with robust standard errors clustered on participants and headlines for Studies 1-4, where we control for the level of education. Robust standard errors (clustered) in brackets. Significance levels: *: p < 0.1, **: p < 0.05, ***: p < 0.01.*

**Duration of the effect**

To check if the effect of the intervention decays over time, here we follow Roozenbeek et al. (2021) and report the main analyses for Studies 2-4^^[[1]](#footnote-1)^^, with an additional control for headline display order, that we interpret as a proxy of time, its two-way interactions with each of the intervention dummies, and its three-way interactions with the intervention dummies and the headline veracity dummy. Table S11 shows that none of the two-way and three-way interactions are statistically significant, suggesting that the effects of the interventions do not significantly decay over time (two-way interactions), not even when we take into account headline veracity (three-way interactions).^^[[2]](#footnote-2)^^

| **Sharing** | **Study 2** | **Study 3** | **Study 4** |
| --- | --- | --- | --- |
| Fake Alert | -0.075***  (0.029) |  |  |
| Accuracy salience |  | -0.101***  (0.038) |  |
| Accuracy endorsement | -0.083***  (0.030) | -0.112***  (0.030) | -0.089**  (0.038) |
| Real | 0.042*  (0.024) | 0.059**  (0.027) | 0.037*  (0.020) |
| Alert x Real | 0.074***  (0.027) |  |  |
| Salience x Real |  | 0.124***  (0.040) |  |
| Endorsement x Real | 0.138***  (0.032) | 0.201***  (0.047) | 0.105**  (0.045) |
| Order | 0.000  (0.000) | 0.001*  (0.001) | -0.002  (0.002) |
| Alert x Order | 0.002  0.001) |  |  |
| Salience x Order |  | 0.000  (0.002) |  |
| Endorsement x Order | -0.001  (0.001) | -0.002  (0.001) | 0.003  (0.004) |
| Alert x Real x Order | -0.002  (0.002) |  |  |
| Salience x Real x Order |  | -0.002  (0.002) |  |
| Endorsement x Real x Order | 0.002  (0.001) | -0.000  (0.002) | 0.003  (0.005) |
| Constant | 0.217***  (0.022) | 0.234***  (0.027) | 0.294***  (0.024) |
| Observations | 13392 | 13200 | 4464 |

**Table S11.** *Linear regression with robust standard errors clustered on participants and headlines (Studies 2-3) or on participants only (Study 4), where we control for headline order and its two-way and three-way interactions with condition and headline veracity. Study 1 is excluded from this analysis because we did not record headline display order. Significance levels: *: p < 0.1, **: p < 0.05, ***: p < 0.01.*

Note that this analysis looks only at the linear decays over time. As noted by Roozenbeek et al. (2021), the decay of the effect need not be linear over time. Following these authors, we thus plot the mean sharing intentions over time, by treatment and headline veracity. We focus only on the main treatments: *baseline* vs *accuracy endorsement*. Figure S4 reports Study 2 and Study 3 (pooled together) and Figure S5 reports Study 4 (we cannot plot it together with Studies 2-3, because Study 4 uses a different number of news headlines). Overall, the effect of the intervention seems to be relatively stable over time, as the difference between the mean sharing in the *accuracy endorsement* and the mean sharing in the *baseline* does not seem to consistently depend on the headline display order.


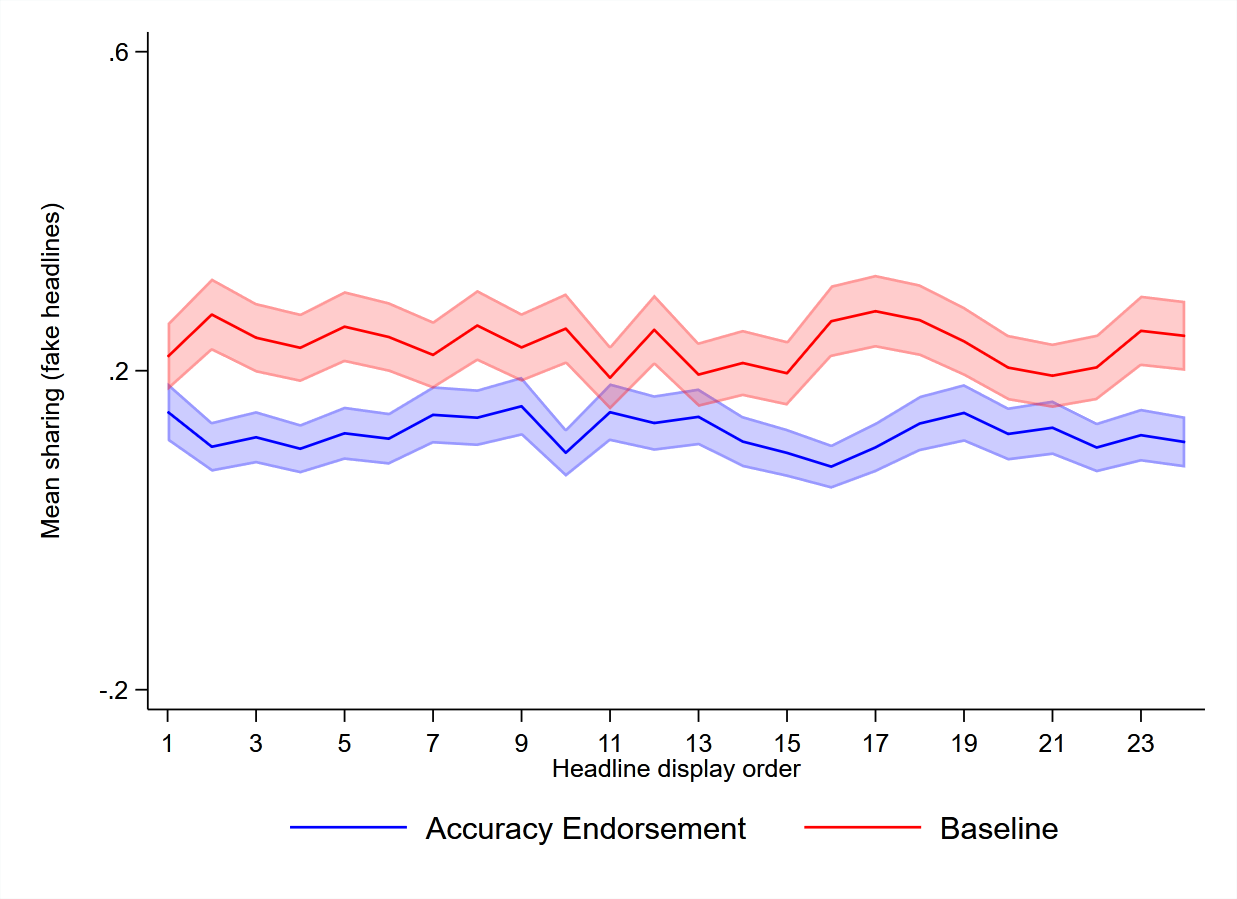

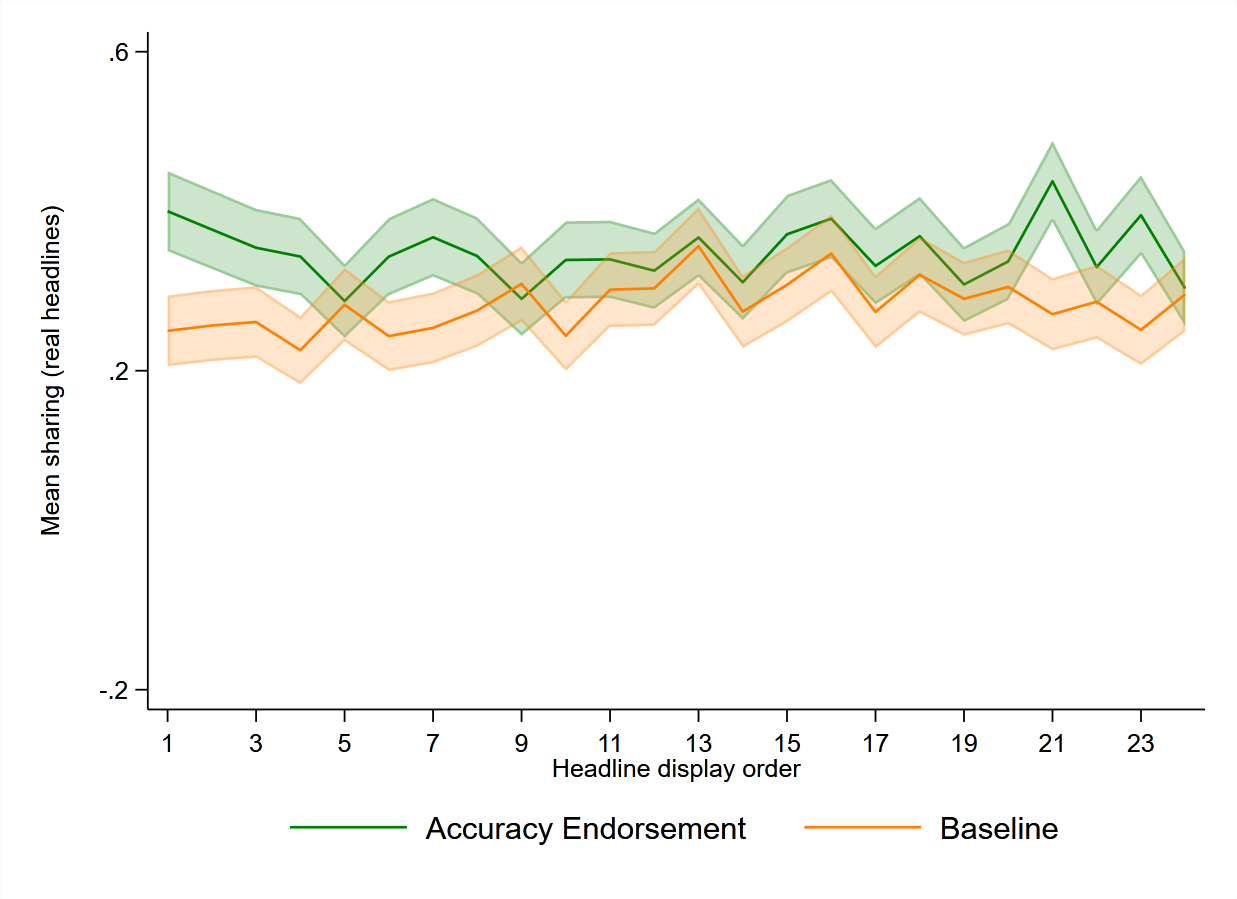


**Figure S4.** *Mean ratings for “sharing intention” of fake news (left) and real news (right) by headline display order for the pooled sample of Study 2 and Study 3.*

*
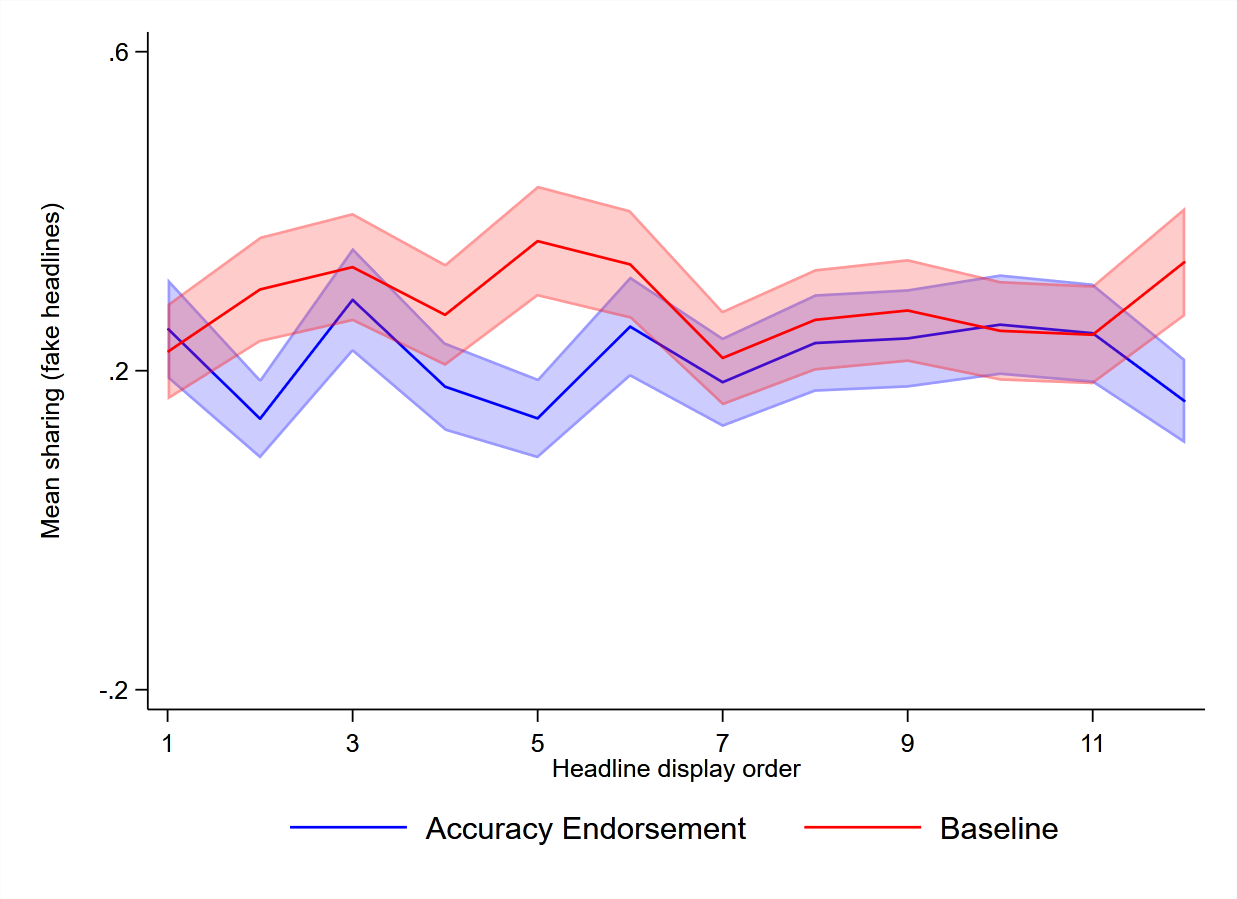

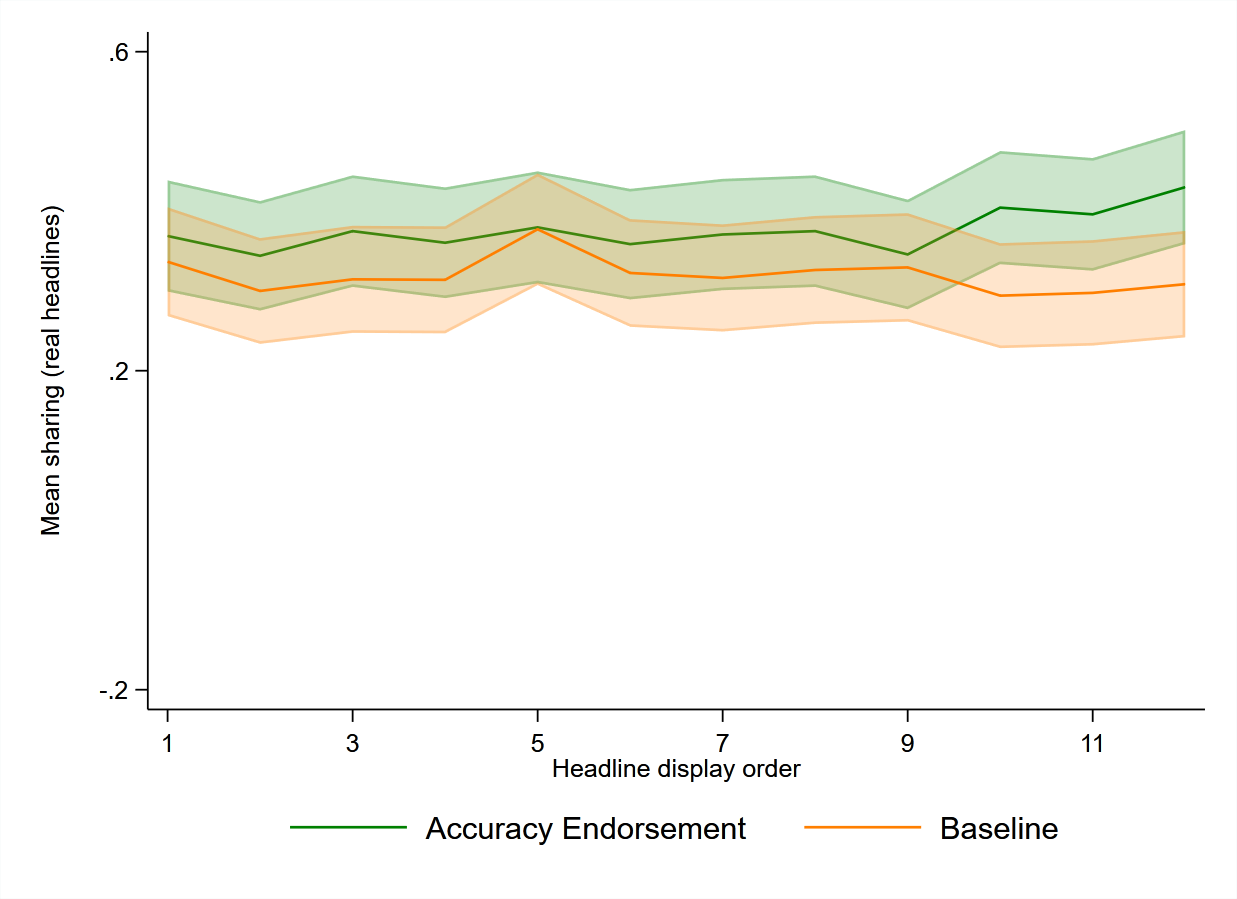
*

**Figure S5.** *Mean ratings for “sharing intention” of fake news (left) and real news (right) by headline display order for Study 4.*

**Excluding headlines that contradict lived experience**

To check if the intervention simply works by making people more likely to pay attention to headlines that contradict their lived experience, here we report an additional analysis to test whether the results are robust to excluding headlines that contradict lived experience.

In Studies 1-3, we eliminate five (out of twelve) headlines:

- Flight 914: Disappeared and landed 37 years later
- Scientists say that a tribe of apes in West Africa are close to develop their own Internet
- Wikileaks alien interview: Death does not exist
- Scientists warn pink cows on the verge of extinction
- Constitution comes forward to accuse Biden of assault

We keep the following headlines:

- COVID-19 found in toilet paper
- Experts say millennials are inhaling too much oxygen
- Oregon fire dept in hot water after first responders kill unarmed man with hose
- Student fed semen filled cupcakes to kids that bullied her
- Man tried to trade kidnapped baby for 15 Big Macs at Arkansas MacDonald’s
- Parents disguising kids as illegal immigrants so they can receive in-person teaching.

In Study 4, we eliminate one (out of six) headline:

- New Jersey brother and sister allowed to marry after 10-year-long court battle

We keep the following headlines:

- Scientists say majority of Earth’s wildlife moving towards more sustainable plant-based diet
- Taliban pledge to allow women and girls as young as ten to perform public execution
- Former “air bud” cast member says he was sexually assaulted by stunt dog
- Jen Psaki: The Biden administration is “in regular touch with” big tech to censor information
- Masks making children more stupid… 23% cognitive development drop

The results are reported in Table S12. The key interaction Endorsement x Real remains highly significant in all Studies 1-4. Moreover, their coefficients are very close to the original ones (Study 1: 0.214 vs 0.239; Study 2: 0.151 vs 0.156; Study 3: 0.195 vs 0.196; Study 4: 0.119 vs 0.126) thus suggesting that the accuracy endorsement intervention does not simply work by making people more likely to pay attention to headlines that contradict their lived experience.

| **Sharing** | **Study 1** | **Study 2** | **Study 3** | **Study 4** |
| --- | --- | --- | --- | --- |
| Fake alert | -0.013  (0.029) | -0.056**  (0.027) |  |  |
| Accuracy salience |  |  | -0.094***  (0.027) |  |
| Accuracy endorsement | -0.051*  (0.028) | -0.084***  (0.025) | -0.132***  (0.027) | -0.065**  (0.028) |
| Real | 0.208***  (0.038) | 0.029  (0.026) | 0.052*  (0.031) | 0.031  (0.054) |
| Alert x Real | 0.011  (0.031) | 0.055**  (0.022) |  |  |
| Salience x Real |  |  | 0.096***  (0.028) |  |
| Endorsement x Real | 0.214***  (0.039) | 0.151***  (0.025) | 0.195***  (0.032) | 0.119***  (0.025) |
| Constant | 0.207***  (0.024) | 0.231***  (0.023) | 0.256***  (0.028) | 0.290***  (0.032) |
| Observations | 10450 | 10602 | 10450 | 4092 |

**Table S12.** *Linear regression with robust standard errors clustered on participants and headlines for studies 1-4, where we exclude news headlines that contradict people’s lived experience. Significance levels: *: p < 0.1, **: p < 0.05, ***: p < 0.01.*

**Supplementary references**

Berinsky, A. J., Margolis, M. F., & Sances, M. W. (2014). Separating the shirkers from the workers? Making sure respondents pay attention on self‐administered surveys. *American Journal of Political Science*, *58*, 739-753.

Horton, J. J., Rand, D. G., & Zeckhauser, R. J. (2011). The online laboratory: Conducting experiments in a real labor market. *Experimental Economics*, *14*, 399-425.

Litman, L., & Robinson, J. (2020). *Conducting online research on Amazon Mechanical Turk and beyond*. Sage Publications.

Pennycook, G., & Rand, D. G. (forthcoming). Nudging social media sharing towards accuracy. *The Annals of the American Academy of Political and Social Science, 25*, 388-402.

Roozenbeek, J., Freeman, A. L., & van der Linden, S. (2021). How accurate are accuracy-nudge interventions? A preregistered direct replication of Pennycook et al. (2020). *Psychological Science*, 09567976211024535.

1. Study 1 is excluded from this analysis because we did not record headline display order. [↑](#footnote-ref-1)
2. In Study 2 and Study 3 we used linear regression with robust standard errors clustered on participants and headlines. In Study 4, this analysis returns no output, because the variance-covariance matrix appears to be non-positive semidefinite. Therefore, we opted to conduct a linear regression with robust standard errors clustered only at the participant level. Note that in this case the standard errors tend to be smaller than with a two-dimensional clustering, so the order effects, that are not statistically significant with a one-dimensional clustering, remain not statistically significant also with a two-dimensional clustering. [↑](#footnote-ref-2)
